# Development of health information materials on antimicrobial resistance with lay workers in Grahamstown/Makhanda, South Africa

**DOI:** 10.3389/fpubh.2025.1542448

**Published:** 2025-10-29

**Authors:** Samridhi Sharma, Sunitha Srinivas, Roman Tandlich

**Affiliations:** ^1^Disaster Management and Ethics Research Group, Faculty of Pharmacy, Rhodes University, Makhanda, South Africa; ^2^Research Associate Disaster Management Division, Stenden South Africa, Port Alfred, South Africa

**Keywords:** antimicrobial resistance, communicative ecology, community healthcare workers, health information materials, health promotion, Makana municipality, peer educators, readability

## Abstract

**Introduction:**

Information design and the design process is vital as part of the health communication strategy to tackle and prevent antimicrobial resistance. Various methods have been developed to achieve holistic tackling of antimicrobial resistance. In primary healthcare and low-resource settings, community healthcare workers and end-user participation allow for interventions to be more effective in meeting the target population’s demands and needs.

**Methods:**

During this study, an antimicrobial resistance health information leaflet and a trainer’s manual were designed in Makana Local Municipality’s primary healthcare settings. The developed materials were assessed for readability using seven readability formulas and suitability using the Patient Education Materials Assessment Tool and the Suitability Assessment of Materials instrument.

**Results:**

The health information leaflet scored a final readability of grade 14, classifying it as ‘difficult’ to read because some medical terms could not be substituted. However, due to written and verbal explanations provided, the community healthcare workers and pharmacist assistants found it easy to understand the health information leaflet and requested no further changes. The finalized health information leaflet obtained a Patient Education Materials Assessment Tool understandability score of 92%, Patient Education Materials Assessment Tool actionability score of 97%, and a Suitability Assessment of Materials instrument score of 91%, proving that it was suitable for its target population.

**Discussion:**

The workshops and trainer’s manual resulted in a significant increase in the peer educators’ antimicrobial resistance-related knowledge. The participants felt empowered and prepared to be the change agents amongst their peers and communities because of the collaborative approach used in the study. The health information leaflet and trainer’s manual on antimicrobial resistance can come in handy for the community healthcare workers and peer educators to use as resources for future home visits and awareness raising campaigns.

## Introduction

Since the World Health Organization (WHO) developed the five strategic objectives of the Global Action Plan on Antimicrobial Resistance (AMR) (1), several new global initiatives in National Action Plans in 194 Member States of WHO are increasingly focusing on “Awareness, Behaviour Change and Education” (2–7). These initiatives are being driven under the first strategic objective to “improve awareness and understanding of AMR through effective communication, education and training”. In 2015, WHO carried out a global public awareness survey on AMR which highlighted the lack of public knowledge on the appropriate use of antibiotics as well as the lack of public understanding of individual behaviour towards the possible development of AMR (5, 6). Along with WHO, the O’Neill report stresses the importance of conducting public-focussed AMR awareness interventions, although it does not provide detailed means of delivering such interventions (6, 8), especially in low- and middle income countries (LMICs).

AMR is partly driven by public knowledge, beliefs, attitudes, and expectations as these factors impact self-medication amongst communities in LMICs where the possibility of access to antibiotics without prescriptions is more likely. Additionally, patient pressure could also be one of the factors influencing prescribing patterns amongst medical doctors (5, 9). Furthermore, non-adherence to antibiotics increases the development of AMR. A study showed that the development of an educational leaflet significantly improved adherence to short-term antibiotics (10). Evidence-based health information leaflets (HILs) as a part of multifaceted interventions are progressively becoming important in influencing end-user knowledge and expectations in antibiotic prescribing, and thus possibly preventing the development of AMR (11).

Written health information can be advantageous not only for those who find it difficult to understand and remember verbal instructions and when counselling may not be possible due to lack of time (12, 13), but for most people as a complementary advantage to verbal instructions. HILs and trainer’s manuals can also be referred to when required to serve as a reminder for end-users as well as their families and caretakers. However, people with low- to semi-literacy skills, particularly in LMICs, may struggle to read and understand most health information (14, 15). This could be due to the high use of scientific and medical terms that could result in misinterpretation and negligence of the given information when the reading materials have not been designed to align to the literacy abilities of end-users. Thus, it is vital to obtain the correct readability level of the given health information based on the readability levels of the target population (16).

A study on AMR conducted in three Thai villages, a rural middle-income setting, concluded that the use of technical medical language is inappropriate for public communication purposes (2). Health interventions which involve the end-users in developing the health information materials have proven to be successful. End-user participation improves the suitability of written materials to meet the participants’ literacy requirements (17, 18). With that being said, it is important to note that minimal studies have engaged with low- to semi-literates when it comes to the design process of written health information materials (18), thus there is an the increased need to conduct such studies.

Health communication can help to bridge the gap between healthcare professionals and communities which lack awareness and knowledge (19, 20). In terms of AMR, the communication strategy needs to capture a complete scope and settings/environment where AMR develops and where its new characteristics emerge. Health communication can further assist in strengthening primary healthcare (PHC) in South Africa by promoting health and preventing diseases (21, 22). Sharma et al. (23) showed that the local conditions in the PHC settings in South Africa, specifically in Makana Local Municipality, are not conducive for counselling of patients about AMR and its development. At the same time, results of the study indicated that there was a lack of training opportunities for the formal and community healthcare workers (CHWs) (23). Using the principles of communicative ecology, Sharma et al. (23) proposed to involve all relevant stakeholders in the PHC settings. That study was the basis of the collection of the data on design of health promotion materials. The study revealed that the factors that control/influence the development of AMR are routine, as reported by the other authors in the literature (23). Lack of training of healthcare workers about AMR and the lack of time to counsel patients about AMR by pharmacists, prescribers and CHWs form a communication gap in AMR management in Makana Local Municipality. This communication gap is the result of lack of information and the contact between the healthcare workers and patients. It is a gap in the context of Luhman’s theory of communication (24) and the framework of communicative ecology (23).

Health communication along with community engagement allows for comprehensive and sustainable health development (22). Raising awareness on the prevention and control of AMR provides an opportunity for further engagement and collaboration between stakeholders (25, 26). Due to the lack of trained healthcare professionals, task shifting with trained healthcare workers, particularly CHWs, are being recognised in many LMICs for delivering PHC services (27, 28). CHWs can therefore reduce the burden on under-resourced and overutilized healthcare systems and increase provision of literacy appropriate health communication, particularly in remote areas. CHWs can effectively contribute towards community outreach programmes and local health promotion, thus assisting in addressing the health equity gap and further supporting the health-related SDGs and Universal Health Coverage (28–30). Peer educators trained for becoming change agents can similarly contribute towards health communication by raising awareness and influencing their peers, families and communities (31–34). These two groups, namely CHWs and peer educators have similar mandates but also slightly different background of recruitment, or the entity they are responsible to. In the current article, these overlapping and yet differing mandates/lines of accountability will be presented as appropriate in a different sections of the article. Such overlaps or differences should be understood by the reader as an expression of the reality on the ground in Makana Local Municipality in combatting AMR. The overlaps and differences are also to be seen as part of the development of the health promotion materials for the specific conditions in the Makana PHC system and in the related settings in the municipality.

This study is part of a strategy to develop a context-specific and culture-sensitive HIL and trainer’s manual on AMR based on a community-based participatory research (CBPR) approach (35) and health communication (19, 20). Settings of this study is Makana Local Municipality, where the human development index increased from 0.57 in 2001 to 0.67 in 2012 [Makana Local Municipality (36), page 64, Figure 7]. Previous studies by the authors and other researchers in the area showed that AMR is an environmental problem (37). Up to 45% of the Makana population did not have any income around the study period [Makana Local Municipality (36), section 2.3.1 page 71 table 38]. Sharma et al. (38) also reported that the most procured antimicrobial therapeutic agent in Makana PHC sector was isoniazid. This indicates that the treatment of tuberculosis was a significant part of the activities in the PHC sector in Makana Local Municipality, i.e., tuberculosis contributes significantly to the burden of disease in the municipality. Significant part of the household income was shown to be required by the Makana population to be spent on the procurement of potable water from alternative resources (39). Dowse et al. (40) reported that the population in South Africa often faces low health literacy levels and so pictograms are a good tool in the engagement in health promotion. Finally, concurrent articles from this research have indicated that the Makana population faces transport barriers in accessing PHC and that this can contribute to the development of AMR in the municipality. It is against this background that the current study seeks to develop a set of HILs and health promotion information manuals to tackle AMR in Makana Local Municipality. The principles of communicative ecology and the aim to understand factors, which control the emergence of AMR in the Makana healthcare system, are driving the material development (23).

## Method

### Research process for health information materials

**Figure 1:**
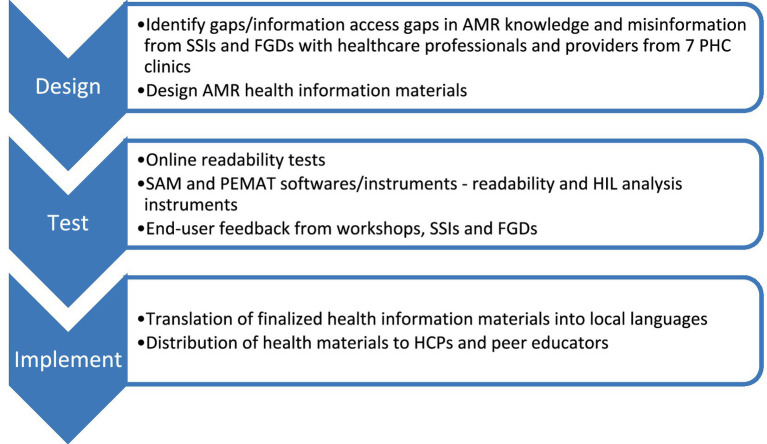
Research process for health information materials. The following abbreviations are used in the figure: SSIs (semi-structured interviews), FGDs (focus group discussions), AMR, SAM, PEMAT, HCPs (healthcare professionals).

Figure 1 shows the research process that took place for the health information materials. In the rest of the article, tables and figures, SSIs stands for the semi-structured interviews, FGDs represent focus group discussions and CHWs stands for the community healthcare workers.

### Development of the HIL

**Figure 2:**
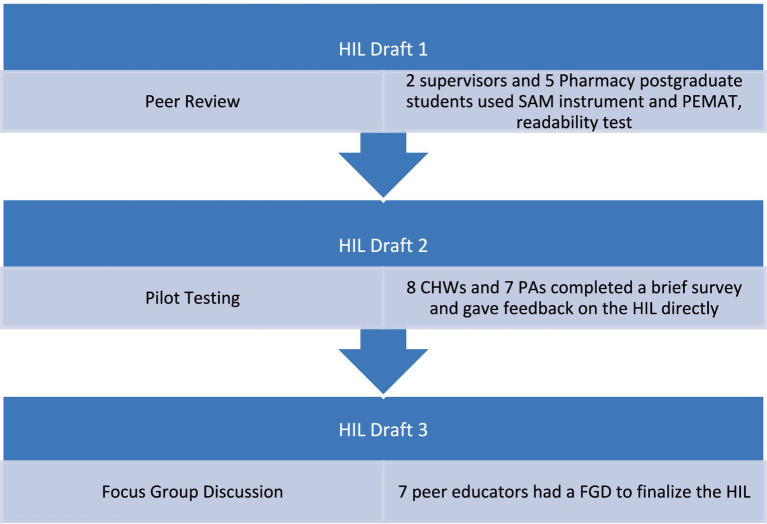
Developmental process of the health information leaflet (HIL). The following abbreviations are used in the figure: patient education materials assessment tool (PEMAT) and the suitability assessment of materials (SAM) instrument, focus group discussion (FGD).

Figure 2 shows the developmental process of the HIL. SSIs and FGDs were conducted with healthcare professionals and providers from seven PHC clinics in Grahamstown/Makhanda, South Africa, to identify the information gap. The information obtained was used to guide the development of a HIL titled “*Antimicrobial Resistance*”. The HIL was first developed in the English language as an A4-sized 3-panel leaflet using Microsoft Publisher and Microsoft PowerPoint. The graphics were either designed manually or obtained from the ‘Creative Commons’ feature on the above-mentioned softwares. Peers from the Faculty of Pharmacy at Rhodes University, and representative groups of the intended users (CHWs and pharmacist assistants—PAs) were consulted regarding the content, graphics, and format of the HIL during the developmental process. Finally, a FGD was conducted with peer educators from Rhodes University to discuss the finalized HIL and to reaffirm if further edits were required.

### Evaluation of the HIL

The readability of the HIL was assessed using seven readability formulas: the Flesch Reading Ease Formula; the Flesch–Kincaid Grade Level; the Fog Scale; the Simple Measure of Gobbledygook (SMOG) Index; the Coleman-Liau Index; the Automated Readability Index; and the Linear Write Formula (41). The Flesch–Kincaid and the SMOG indices indicate what level of education or engagement with the topic the reader must achieve for comprehension of the message that is being communicated. The full text of the HIL was copied and pasted into the online readability tool which automatically assessed the seven readability scores using the above-mentioned formulas as well as the average readability score. The quality and suitability of the HIL was assessed using the PEMAT (42), and the SAM instrument (43, 44).

The PEMAT is a systematic method intended to be completed by professionals, including health care professionals and providers, to assess the “understandability” and “actionability” of health information materials. “Understandability” allows users with varying demographics and health literacy levels to easily comprehend and explain key messages, whilst “actionability” allows them to easily identify what behaviours and/or activities they can implement based on the information provided. The PEMAT rates health information materials in the following seven topics:

1. Content
2. Word choice and style
3. Use of numbers
4. Organization
5. Layout and design
6. Use of visual aids
7. Actionability

The PEMAT has a total of 26 items with the response options of ‘Disagree’ where a score of 0 is given or ‘Agree’ where a score of 1 is given. Some items also have a response option of ‘Not Applicable’. An item is rated as “Agree” when it is met 80–100% of the time throughout the health information materials. The PEMAT provides one score for “understandability” and a separate score for “actionability” where the scores are calculated as shown in Equations 1, 2 (42):


(1) $$\begin{array}{l} \text{PEMAT Understandability Score}\;(\%)\\ =\frac{\text{Total points for}\prime \text{understandability}\prime }{\text{Total possible points for}\prime \text{understandability}\prime }\times 100 \end{array}$$

(2) $$\begin{array}{l} \text{PEMAT Actionability Score}\;(\%)\\ =\frac{\text{Total points for}\prime \text{actionability}\prime }{\text{Total possible points for}\prime \text{actionability}\prime }\times 100 \end{array}$$

The SAM instrument is a systematic method to quantitatively evaluate the suitability of health information materials, including the readability and cultural appropriateness of the materials. The SAM instrument rates health information materials in the following six factors:

- Content
- Literacy demand
- Graphic illustrations, lists, tables, charts
- Layout and typography
- Learning stimulation and motivation
- Cultural appropriateness

The SAM instrument has 22 items in total with the response options of ‘superior’ where a score of 2 is given, “adequate” where a score of 1 is given, or “not suitable” where a score of 0 is given. The SAM score is calculated as shown in Equation 3 (44):


(3) $$\mathrm{SAM}\;\text{Score}\;(\%)=\frac{\text{Total}\;\mathrm{SAM}\;\text{score}}{\text{Total possible score}}\times 100$$

Two supervisors and five postgraduate students from the Faculty of Pharmacy, Rhodes University provided feedback on the first draft of the HIL using the PEMAT and SAM instruments and provided additional comments/suggestions. The PEMAT and SAM scores were compared, and the feedback was used to finalise a second draft of the HIL. Eight CHWs and seven PAs from the seven PHC clinics volunteered to participate when approached with the Participant Invitation Letter. They were selected by means of stratified random sampling – ensuring that there is at least one participant from each PHC clinic – with the assistance of the District Pharmacist at the Eastern Cape (Makana Sub-District) Department of Health and were required to sign the Participant Informed Consent Form before the pilot testing commenced. Participants were requested to provide their demographic details. The second draft of the HIL was pilot tested using a brief survey (see the Supplementary information) and a copy of the HIL to ensure its suitability and comprehension amongst the target users. Participants were asked to identify and circle text on the HIL which they did not understand, comment on the culture-sensitivity and appropriateness of the text and illustrations used and provide feedback on how the HIL can be improved. Remarks made by the participants were compared, and the feedback was used to compile the third draft of the HIL. Most participants’ feedback was similar, i.e., data saturation was reached; hence the testing did not continue.

### Development of the trainer’s manual

**Figure 3:**
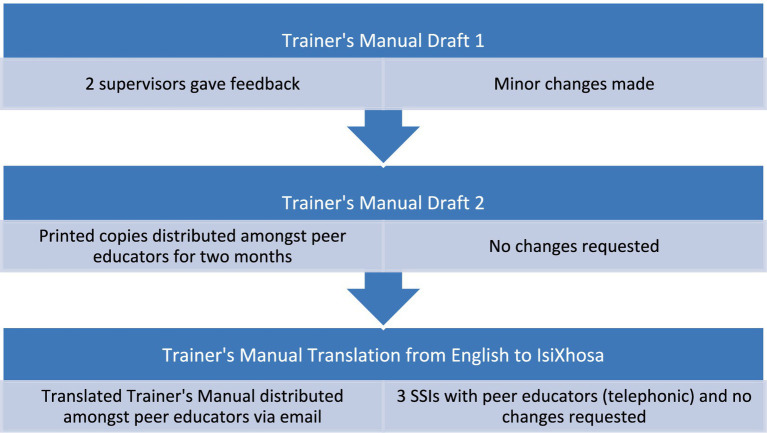
Developmental process of trainer’s manual. The abbreviation of SSIs stands for semi-structured interviews.

Figure 3 shows the developmental process of the trainer’s manual. During the Rhodes University peer educators’ workshop, the topic of ‘Antimicrobial Resistance’ was introduced to 21 peer educators. The researcher was allocated an hour-long session which was inclusive of a presentation, an activity/demonstration on hand hygiene, and a question-and-answer session. An IsiXhosa interpreter was present throughout the workshop to confirm understandability amongst the peer educators. Once the participants confirmed that they were comfortable to talk in English, the interpreter was not used for future workshops and discussions. The Participant Invitation Letter and Participant Informed Consent Form were distributed to all peer educators prior to the presentation. Upon completion of the consent form, the peer educators completed a pre- and post-workshop questionnaire (see pre- and post-workshop questionnaire for workshop 1 in the Supplementary information) which consisted of multiple-choice questions and Yes/No questions. The purpose of the questionnaire was to quantify whether the workshop was able to achieve its aim of increasing awareness and providing relevant information on AMR.

The presentation and activities from the workshop formed the basis of the first draft of the AMR trainer’s manual. The previously developed HIL was also added to the trainer’s manual. Two supervisors from the Faculty of Pharmacy as well as the peer educators from Rhodes University were consulted whilst developing the trainer’s manual. The trainer’s manual was first developed in the English language as an A4-sized document using Microsoft Word and Microsoft PowerPoint. The graphics were either designed manually or obtained from the ‘Creative Commons’ feature on the above-mentioned softwares. The images used for ‘hand hygiene’ and the instructions regarding ‘how to wash your hands’ were taken by the researcher with the assistance of Pharmacy Practice postgraduates from the Faculty of Pharmacy, Rhodes University.

### Evaluation of the trainer’s manual

Seven peer educators from Rhodes University volunteered to participate in a FGD when approached with the Participant Invitation Letter. They were selected by means of simple random sampling with the assistance of a key stakeholder who coordinates activities of peer educators and were required to sign a Participant Informed Consent Form prior to the FGD. Participants were requested to provide their demographic details. The researcher went through the latest draft of the HIL with the peer educators to reaffirm if further edits were required. This session was also used to discuss the development of the trainer’s manual with the peer educators and to obtain any feedback and/or comments on additional information which the peer educators would like to see in the trainer’s manual. The peer educators again completed a pre- and post-workshop questionnaire (see pre- and post-workshop questionnaire for workshop 2 in the Supplementary information) which consisted of multiple-choice questions and Yes/No questions. The purpose of the questionnaire was to quantify if the peer educators recalled the information covered in the first workshop as well as to reiterate the key points relating to AMR and those discussed in the HIL.

Two supervisors from the Faculty of Pharmacy, Rhodes University provided feedback on the first draft of the trainer’s manual. The minor feedback and comments were used to finalise a second draft. The second draft of the trainer’s manual was printed as an A4-sized document in colour and distributed amongst peer educators with the assistance of a key stakeholder who coordinates activities of peer educators. The peer educators were given a period of two months to go through the trainer’s manual and provide feedback on the manual. Since no changes were requested, the manual was then translated into IsiXhosa and distributed again, via email, amongst the peer educators. At this stage, in-person FGDs and/or SSIs were not conducted due to the COVID-19 protocols and circumstances. After a few months, SSIs were conducted with three peer educators via WhatsApp calls to obtain feedback (see the Trainer’s manual feedback in the Supplementary information) on the finalized trainer’s manual. All three peer educators confirmed that further changes in the trainer’s manual were not required; thus, testing did not continue, and the English and IsiXhosa versions of the trainer’s manual were finalized (see the final Trainer’s manual in the Supplementary information).

### Translation and distribution of the health information materials

The final version of the HIL and trainer’s manual were translated into IsiXhosa and Afrikaans; two local languages of the Eastern Cape Province in South Africa. They were distributed amongst the peer educators at Rhodes University, as well as the healthcare professionals and providers from the seven PHC clinics in Grahamstown/Makhanda with the assistance of the District Pharmacist at the Eastern Cape (Makana Sub-District) Department of Health.

### Social network for stakeholders

**Figure 4:**
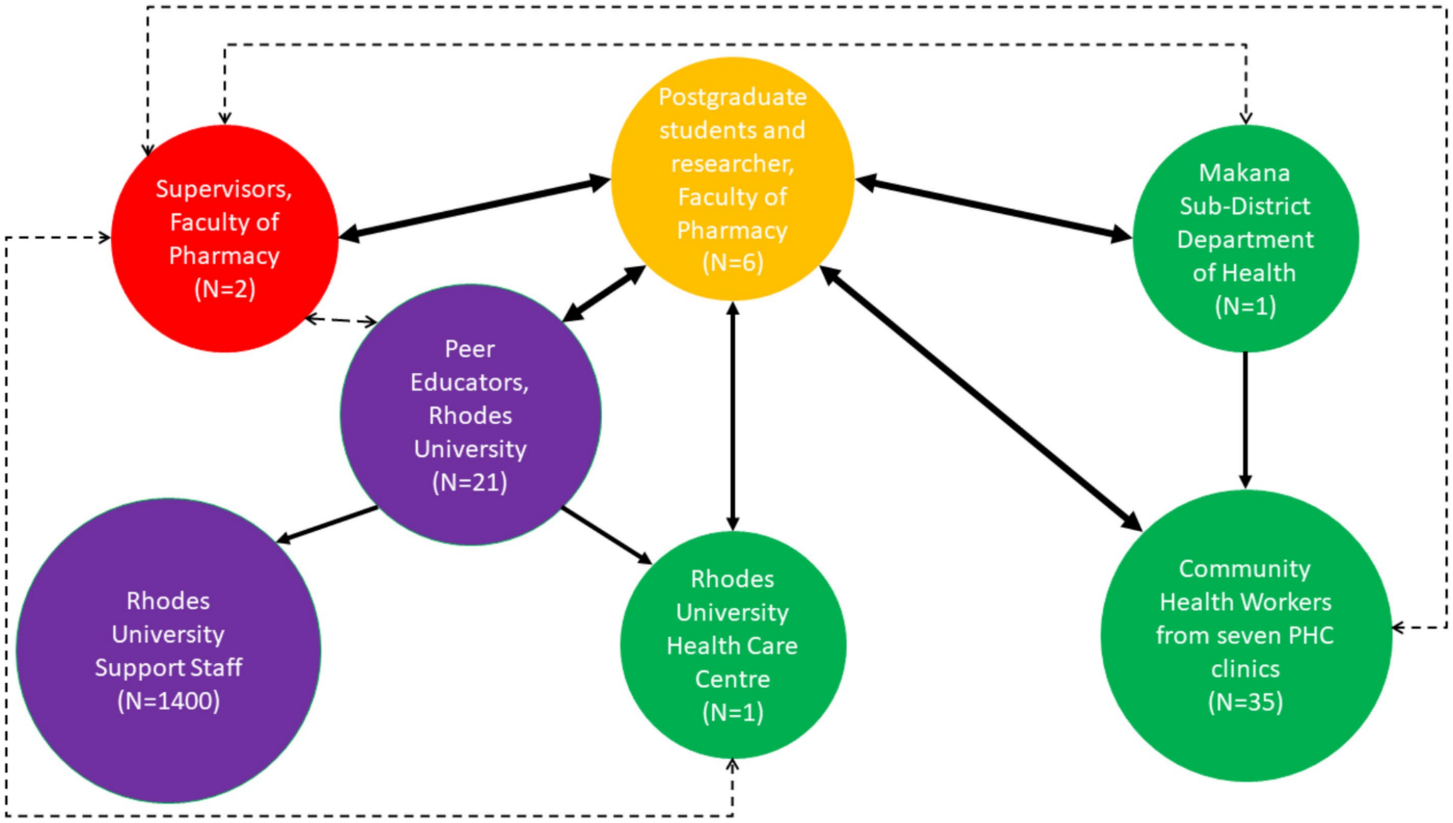
Social network for stakeholders, where PHC stands for primary healthcare.

Figure 4 shows the social network for the stakeholders in this study.

In the below social network, the size of the circle indicates the relative size of the given group, i.e., the bigger the circle, the more people involved. The direction of the arrows indicates the flow of information and direction of communication. The solid lines indicate full interaction as opposed to the dashed lines which indicate partial interaction, i.e., supervision. The thickness of the arrows indicates the amount of interaction which took place during the study. The study participants were recruited based on the previous work that some of the authors of the current paper, as well as other researchers from the Faculty of Pharmacy at Rhodes University, have done with the peer educators, and the CHWs in the Makana health environment (38, 45, 46). The study participants were recruited based on being active CHWs and peer educators at the time of the current study. The approach was convenience and purposive sampling.

From a theoretical point of view, the study approach was rooted in the Luhman’s theory of communication and development of the health promotion materials is context-specific for the Makana healthcare settings. Health promotion materials are created by engagement between the different stakeholders in the PHC clinics and the community members who are at the boundary between the community – patients and the healthcare systems, namely CHWs (as based on the page of the article – see above). By combining the theoretical and global knowledge about the factors that drive AMR development, with local knowledge and specific factors, the authors hoped to develop health promotion materials that would maximise the success of any public health intervention to tackle AMR development and spread in Makana Local Municipality. Tackling AMR requires that project activities, such as those described in the current article, are interactions among humans. Those interactions create communication according to Luhman’s theory of communication. Such interaction can be harnessed to tackle AMR, and the tackling can be facilitated by the group of people who are creating the communication network according Luhman’s theory. Communication occurs through contact and the exchange of information facilitates understanding about the extent of AMR as a public health problem. This information then lays the foundation for tackling AMR.

### Data validity and reliability

Data validity and reliability were ensured by including the researcher’s supervisors, Pharmacy postgraduate students, and peer educators from Rhodes University, as well as at least one PA and one CHW from the seven PHC clinics as participants for this phase of the study. This approach ensured that peer educators from different departments at Rhodes University and all PHC clinics were represented without generalisation and feedback was taken into consideration without bias. Data validity was further strengthened through the use of brief surveys for pilot testing and focus group discussions which were recorded alongside with note-taking. The use of seven online readability tests, and the PEMAT and SAM instrument for testing the health information materials, and end-user feedback from the workshops with peer educators further supported data reliability. Quotations from the qualitative data are presented as direct transcription of the recordings from the data collection part of the study. Where translations were done and are reported on in this article, the language was adjusted to ensure and to reflect the authenticity of the participants quotes as much as possible. Data presented in the Results section is deemed a representative presentation of the interviewees opinions and inputs.

### Ethical clearance

The research project was approved by the Rhodes University Faculty of Pharmacy Ethics Committee (PHARM 2017–05) and extended by Rhodes University Ethical Standards Committee three times under the same tracking number. Permission and clearance to work in the Department of Health facilities was also granted under the tracking number: EC_2017RP24_25.

## Results

### Development of the AMR health information leaflet

The participants’ demographics are shown in Supplementary Table S1. Their age ranged between 29–59 years, with an average of 41 ± 10 years. In total, 14 out of 15 participants (93.33%) were females and IsiXhosa was the home language of all the participants. Results of seven readability tests for the first and third drafts of the HIL are presented in Table 1.

**Table 1:** Readability scores for health information leaflet (HIL) draft 1 and draft 3.

Readability test	Score		Grade level of readability	
	HIL draft 1	HIL draft 3	HIL draft 1	HIL draft 3
Flesch reading ease score	11.6	33.3	Very difficult to read	Difficult to read
Gunning fog	13.9	13.5	Hard to read	Hard to read
Flesch–Kincaid grade level	15.8	13.9	College Graduate and above	College
The Coleman-Liau index	19	13	Graduate college	College
The SMOG index	12.9	11.9	College	Twelfth grade
Automated readability index	16.1	14.2	College graduate	21–22 years old (college level)
Linsear write formula	13.5	14.9	College	College

Using the Past 3.0–5.0 statistical software package (see https://www.nhm.uio.no/english/research/resources/past/ for details, website accessed on 19th July 2025), the Mann–Whitney test at 5% level of significance showed that there was no significant difference between the HIL readability scores for draft 1 and draft 3 (*p*-value = 0.9819). There was, however, qualitative improvement in the readability of the HIL was observed. Summary of the seven readability formulae is summarised as the average readability score of the first and third drafts of the HIL in Table 2. The PEMAT and SAM results of the first draft of the HIL are presented in Table 3. Using Past software, the PEMAT understandability scores for the first HIL draft and the third draft were not statistically significant at 5% level of significance (the Mann–Whitney test, p-value = 0.5245).

**Table 2:** Average readability scores as grade of education for the health information leaflet (HIL) draft 1 and draft 3.

Average readability score	HIL draft 1	HIL draft 3
Grade level	16	14
Reading level	Very difficult to read	Difficult to read
Reader’s age	College graduate	21–22 years old (college level)

**Table 3:** Patient education materials assessment tool (PEMAT) and the suitability assessment of materials (SAM) instrument and results for the health information leaflet (HIL) draft 1.

Reviewer	PEMAT score (%)		SAM score (%)
	Understandability	Actionability	
1	94	100	89
2	100	100	73
3	83	80	81
4	88	100	84
5	71	80	66
Average score + STD	87 ± 11	92 ± 11	79

For the actionability scores, similar testing revealed analogical results (the Mann–Whitney test at 5% level of significance, *p*-value = 0.6825). Finally, the SAM scores were not statistically significant at 5% level of significance, but the testing results were borderline (the Mann–Whitney test, *p*-value = 0.0571).

The PEMAT and SAM results of the third draft of the HIL are presented in Table 4.

**Table 4:** Patient education materials assessment tool (PEMAT) and the suitability assessment of materials (SAM) instrument and results for the health information leaflet (HIL) draft 3.

Reviewer	PEMAT score (%)		SAM score (%)
	Understandability	Actionability	
1	100	100	95
2	100	100	90
3	90	95	95
4	90	100	95
5	80	90	80
Average score + STD	92 ± 8	97 ± 4	91

The first draft of the HIL was modified to create a second draft after the initial peer review. 34 changes inclusive of textual and imagery changes were made to the first draft. Seven of the 34 changes were imagery changes – five images were replaced with more suitable ones, and two were deleted from the HIL. 27 textual changes were made, such as:

*“Low- and middle-income countries are most vulnerable to Antimicrobial Resistance” was rephrased to “Developing countries are more at risk to Antimicrobial Resistance.”*

“Overuse in fish farming and livestock” was rephrased to “Overuse in livestock (e.g., cattle, goat, pig, chicken) and fish farming.”

“Regularly washing your hands” was rephrased to “wash hands regularly with clean water and soap.”

The table of changes from the first draft to the second draft of the HIL is shown in Supplementary Table S4 in Appendix 2. Draft two was modified to draft three after pilot testing. 35 changes, including both textual and imagery changes, were made. Eight of the 35 changes were imagery changes – two images were replaced with more suitable ones, two images were deleted from the HIL, and four new images were added to the HIL to support the text. 27 textual changes were made, for example:

“Inappropriate use, e.g., use of antibiotics for viral infections (colds and flu)” was rephrased to “Using antibiotics when they are not necessary” as one bullet point and “Antibiotics fight infections caused by bacteria. Antibiotics do not work against viruses such as colds and flu” as another bullet point.

“Poor infection control in hospitals and clinics” was rephrased to “Keeping a place dirty.”

The table of changes from the second draft to the third draft of the HIL is shown in Supplementary Table S5 in Appendix 2. Regarding the relation of text to images, participant 1 stated that, “Even a person who has no health education can just look at the picture and understand the message.” However, four participants did not understand what all the pictures illustrated. All participants found the font legible and the amount of text in the HIL to be “just right.” Most of the participants found the language easy to understand. Participant 4 commented, “The wording is simple and straightforward,” and participant 6 commented, “Yes the English that you use is simple and understandable.” However, participant 15 commented, “Not easy to understand because my first language is Xhosa.” Overall, most participants found the HIL extremely helpful. Participant 1 said, “Keep up the good work. Thank you for listening to the CHW demand of trying to understand Treatment Adherence.” Participant 12 suggested, “I think everyone should get this information especially our patients, because they are on antibiotics as soon as they feel better, they stop using them. Also, they do not know the importance of using vaccines as they say vaccines make them sick.” Some participants showed preference for a HIL in IsiXhosa as participant 14 said, “We want Xhosa leaflet.”

### Workshops on AMR with peer educators

The first AMR workshop hosted for the peer educators at Rhodes University included 21 participants, and their demographics are shown in Supplementary Table S2 in Appendix 1. The participants had an average age of 47 ± 9 years, with 17 participants (81%) being females and 4 participants (19%) being males. IsiXhosa was the home language of 20 (95%) of the participants, with English being the home language of one participant (5%). Questionnaire data from the peer educator workshops are shown in Table 5. The second AMR workshop hosted for the peer educators at Rhodes University included seven participants, and their demographics are shown in Supplementary Table S3, and results are presented in Table 6. The participants had an average age of 46 ± 7 years, with 17 participants (81%) being females and four participants (19%) being males. IsiXhosa was the home language of 20 (95%) of the participants, with English being the home language of one participant (5%).

**Table 5:** Pre- and post-workshop questionnaire for peer educators for the antimicrobial resistance (AMR) workshop on 23 May 2019 (*N* = 21).

No.	Question	Multiple-choice options	Pre-workshop questionnaire (*N* = 21)			Post-workshop questionnaire (*N* = 21)		
1	Are all types of microbes harmful?		**Yes** 4	**No** 15	**Blank** 2	**Yes** 2	**No** 19	**Blank** 0
2	What are antibiotics?	[Antibiotics are medicines that help to fight viruses]	2			5		
		[Antibiotics are medicines that help to fight bacteria]	8			10		
		[Antibiotics are medicines that help to fight all microbes]	4			1		
		[Antibiotics do not work]	0			0		
		[Blank]	6			5		
3	Can antibiotics help to fight the flu?		**Yes** 19	**No** 2	**Blank** 0	**Yes** 9	**No** 12	**Blank** 0
4	What is antibiotic resistance?	[Antibiotic resistance is when antibiotics can fight and kill bacteria]	7			6		
		[Antibiotic resistance is when antibiotics cannot fight and kill bacteria]	7			10		
		[Antibiotic resistance is when antibiotics can fight and kill all microbes]	2			1		
		[Antibiotic resistance is when antibiotics cannot fight and kill all microbes]	3			4		
		[Blank]	2			0		
5	What can cause antibiotic resistance?	[Using antibiotics exactly as prescribed by the doctor or nurse]	9			7		
		[Keeping the environment clean]	0			1		
		[Completing the antibiotic course]	3			0		
		[Sharing and using leftover antibiotics]	7			11		
		[Blank]	2			2		
6	Can resistant bacteria be treated with the same antibiotic?		**Yes** 7	**No** 14	**Blank** 0	**Yes** 2	**No** 18	**Blank** 1
7	Can we pick up microbes from the things we touch?		**Yes** 12	**No** 8	**Blank** 1	**Yes** 13	**No** 8	**Blank** 0
8	Can we remove all microbes by washing our hands with water alone?		**Yes** 4	**No** 15	**Blank** 2	**Yes** 3	**No** 18	**Blank** 0
9	When should we wash our hands?	[After using the toilet]	1			0		
		[Before preparing food]	0			0		
		[After coughing or blowing our nose]	0			0		
		[All of the above]	19			21		
		[Blank]	1			0		
10	What will happen if antibiotic resistance keeps increasing?	[There will be more deaths]	15			20		
		[We will no longer get sick]	3			1		
		[We will spend less time in hospitals]	0			0		
		[We will spend less money on medicines]	2			0		
		[Blank]	1			0		
11	How can we tackle antibiotic resistance?	[Get antibiotics as soon as we feel sick, either from the pharmacy or from a friend]	2			2		
		[Share antibiotics with our families when they are sick]	0			0		
		[Only take antibiotics as prescribed by our doctor or nurse]	15			18		
		[Stop taking antibiotics once we feel better]	3			1		
		[Blank]	1			0		

No.	Question	Post-workshop questionnaire (*N* = 21)
12	Do you have any further comments and/or suggestions?	“Antibiotic is very useful to our life; sometimes it used to kill bacteria around the body, and the antibiotic is good for using to our bodies”
		“No I do not have any comments, but I learn so much”
		“I learn a lot because get antibiotic to the doctor and not finish sometime and I took it again and learnt that must not do that”
		“My comments in this point are centred around—thanks for let us know about these thing so that we may have South African people stay alive and be healthy.”
		“I’ve got interested information about antibiotic I can use to tell people and can also help me.”
		“I learn a lot because I use to share with my families and leave when I feel sick eat again”
		“Antibiotics is a dangerous thing if used wrongly. It can be the cause for more illness. It is not always helpful should be used or prescribed and when necessary.”
		“I have learnt that antibiotics are not needed for flu but sometimes when I visit the doctor or nurse I do get antibiotics for flu, and I do use them as prescribed which leaves me a bit confused on the flu issue.”
		“Medication needs to be taken when necessary to avoid being resistant to meds. Do not take it if you do not need to. Take it when it is prescribed by a medical professional.”
		“Sharing antibiotics is not right or when you feel better stop using them and then when you sick you go back to what you have left.”
		“1. I do not trust doctors and nurses anymore!! 2. Thank you for sharing, at least now I will know to refuse them when they are given in unnecessary cases like flu”
		“The session was educational and good.”
		“I learn more that I know”
		“[BLANK]”
		“People must stop using an antibiotic without distribute by doctor or nurse, when the doctor prescribed you must do so.”
		“[BLANK]”
		“Washing hands with soap and water; keep all environments clean and germ free. Thank you for your presentation!”
		“No. Thank for the information”
		“If you do not have clean water or soap can use waterless hand sanitizer.”
		“It is wise to tell the doctor or nurse when you last did take antibiotics before you get these new ones from doctor or nurse. That is helping to check the period between the times you use antibiotics.”
		“Thank so much Sam for your lesson. I’ve learned a lot as a results I will share the information to others”

The correct answers were highlighted in green for both, pre- and post-workshop questionnaires.

**Table 6:** Pre- and post-workshop questionnaire for peer educators for AMR HIL workshop on 23 October 2019 (*N* = 7).

No.	Question		Pre-workshop questionnaire (*N* = 7)		Post-workshop questionnaire (*N* = 7)	
			Yes	No	Yes	No
1	Antimicrobial resistance is when the correct medicine can no longer fight the infection.		4	3	6	1
2	Antimicrobial resistance only occurs in patients over 65 years.		1	6	1	6
3	What will happen if antibiotic resistance keeps increasing? (Multiple-choice question)	[There will be more deaths]	4		4	
		[We will not get sick]	2		2	
		[We will spend less nights staying in the hospital]	0		0	
		[We will spend less money on medicines]	1		1	
4	What can cause antibiotic resistance? [Using the antibiotics exactly as prescribed by the doctor or nurse at the clinic]		5	2	5	2
	What can cause antibiotic resistance? [Finishing the antibiotic course]		4	3	4	3
	What can cause antibiotic resistance? [Sharing antibiotics with others]		3	4	3	4
	What can cause antibiotic resistance? [Using leftover antibiotics]		3	4	3	4
	What can cause antibiotic resistance? [Keeping the place clean]		4	3	5	2
5	Can we remove all harmful microbes by washing our hands with water alone?		2	5	1	5
6	We should wash our hands before __________ [using the toilet]		3	4	3	4
	We should wash our hands before __________ [cooking food]		7	0	7	0
	We should wash our hands before __________ [coughing]		3	4	3	4
	We should wash our hands before __________ [blowing our nose]		3	4	3	4
7	How can we control and manage the increasing antibiotic resistance? [Get antibiotics from the clinic as soon as we get the flu or cold]		5	2	1	6
	How can we control and manage the increasing antibiotic resistance? [Stop taking antibiotics once we feel better]		3	4	2	5
	How can we control and manage the increasing antibiotic resistance? [Share antibiotics with our families and friends when they are sick]		1	6	0	7
	How can we control and manage the increasing antibiotic resistance? [Using the antibiotics exactly as prescribed by the doctor or nurse at the clinic]		7	0	6	1

No.	Question	Pre-workshop questionnaire (*N* = 7)	Post-workshop questionnaire (*N* = 7)
8	Do you have any further comments and/or suggestions?	“No”	“No not at all”
		“I like to be part of this because is where I increase my knowledge and understanding of antibiotics”	“Eat treatment regular.”
		“I and you are using the direction exactly you can get better for your everything more especial healthy.”	[Blank]
		[Blank]	“Yes the clinics must tell us if you do not finished your medicine you will not get better at all”
		“No I do not have any question so far”	“We have learned a lot about antibiotics and can be helpful to our families and friends”
		“The correct use of antibiotics is at our advantage and very helpful – but the incorrect use of them can cause your death. Thank you for teaching us all of this. I am now more conscious about using antibiotics. I can also apply this at home and at my workplace. I can also help my children.”	[Blank]
		“More awareness, sharing with family and friends, talking about antibiotic myths”	“I learned a lot from this section about antibiotics”

The correct answers were highlighted in green for both, pre- and post-workshop questionnaires.

### Feedback from peer educators on the AMR trainer’s manual

In the first FGD focusing on the first draft of the AMR trainer’s manual, the peer educators requested for some words to be simplified further with the addition of more pictures. They had also requested for the hand washing instructions to be better explained with the use of images instead of “text only.” Feedback on the second draft of the AMR trainer’s manual was obtained through SSIs via WhatsApp calls with three peer educators.

### Collaborative research approach and build-up to the trainer’s manual

The collaborative approach used in developing the health information material with the target group instead of for the target group had a positive impact, which is evident from the responses made by the peer educators during their final semi-structured interviews, as shown below:

*“Yes, yes, it [the build-up to the trainer’s manual] has been very helpful, and we also appreciate the fact that we were actually involved contributing for the manual.”* (Participant number 1 – peer educator)

*“It [being involved in the whole developmental process] helps firstly on a personal level. It helps with the understanding and the fact that we felt valued because we put in the input and at the same time, we also felt the sense of belonging because this was something that was started from scratch and where we were involved. So, we felt this was a manual that was being built and being made for us.”* (Participant number 2 – peer educator)

*“Something that you are confident about and something that you have been involved in, you could explain it to others, instead of having this big book in front of you with all these big jargon words which you cannot also understand.”* (Participant number 3 – peer educator)

### Helpfulness of the trainer’s manual

The trainer’s manual presented new and important information to the peer educators, as indicated by some of their responses below:

*“I always thought that if I go to a doctor for fever or if I feel something, I’ve always had this expectation of if I do not get antibiotics or I do not get an injection, it’s like I will not get better quicker… That I need to go to a doctor, then if I go to a doctor, I expect to get antibiotics. So, this manual has actually helped me in informing me that not every illness requires you to have [antibiotics]. A mere fever does not necessarily require me to get antibiotics.”* (Participant number 1 – peer educator)

*“If I go and I get some antibiotics for a certain thing, throat infection or something, and my sister will experience the same thing maybe two weeks after, then I can say I still got my antibiotics, which I got from the doctor, I never got to finish them. Because the moment I feel better, then I will just leave the pills. Meanwhile, it is written on most of the antibiotics, they say finish them, finish the pills but we’ll always leave that too… Then now we have got this, now if my sister feels the same thing, then I would say, you know, sort of like sharing it. And I’ve picked up that I need to finish the antibiotics every time when I get them.”* (Participant number 2 – peer educator)

### AMR and COVID-19

The trainer’s manual was also very handy given the context of COVID-19, and the following responses from the peer educators highlighted this further:

*“It will help a lot, Sam because if you look at the manual, especially if you look at round about page 11, then page 12, going into that activity at page 13, it’s all basically about hygiene. And it explains how you can wash your hands, which is something that has become a norm now, you know, with the COVID… And also, the sentences [to] avoid close contact, the social distancing now that we are doing due to COVID… Practicing safer sex, trying to stay away. Now even if you are a married couple, to stay safe there are instances whereby you have to be in different bedrooms, and even at home with the whole family. Now you know, hygiene is like a first priority.”* (Participant number 2 – peer educator)

*“When I look at your manual on that page 13, where it’s got this activity… Since you know the COVID started it was wash your hands with soap… When I look at that activity, it explains to us the importance of having that dishwashing liquid, and I like the fact that under materials, you mentioned dishwashing liquid because it’s something like every home that has got. It’s not something that’s specific that you have to go out and go buy, you know, which you cannot afford, its soap, it’s something that everyone can have around. And it also explains the importance of having that soap in your hands because if you are just doing it under a tap with water, you are not washing away the germs.”* (Participant number 2 – peer educator)

*“I think yeah, the manual is very useful and also note the fact that on the next page it goes on food as well. So, it does help us with the COVID as well because not only are you doing this at the workplace, but you are also doing this at home as well… We can also teach our children these things because now the COVID has opened that communication where we can teach even our children and our family members more about hygiene, you know, our [older] mothers more about cooking when they are in the kitchen, to preserve these things, and to keep hygiene a high priority.”* (Participant number 3 – peer educator)

### Translation of the trainer’s manual

The second draft of the trainer’s manual, which was presented in both English and IsiXhosa, was very well received by the peer educators, as shown in their responses below:

*“Now I see on this one that you do the English then you do the IsiXhosa version. I’m very happy to see that, because, as you know, we are peer educators on this staff, and some of our staff members are quite [older] people, so some of them, they will not understand certain things [in English].”* (Participant number 1 – peer educator)

*“Even though we are Xhosa speaking people, but I guess when you are at work, the medium you use for the whole day is English. Sometimes you will find this word is in your head, but you cannot actually quickly translate it to IsiXhosa. So, I think that will help us as well because it will sort of give us a guideline to our own language what this [manual] is saying.”* (Participant number 2 – peer educator)

### Format of the trainer’s manual

The format of the trainer’s manual was highly appreciated by the peer educators, as shown in their comments below:

*“I like the way that it’s framed out because it gives us what to expect per page, everything is outlined. And then it’s got this preference [preface] as well that’s there giving us what the manual is supposed to do… That it’s supposed to help us, you know, into creating awareness.”* (Participant number 1 – peer educator)

*“When you are giving the information, you do give us like sort of like a background, what we can expect to learn from this manual, and also when it’s giving us that information, it’s got these nice pictures we can look at… and the formatting of the manual. I really like it, and I think it’s easy to understand.”* (Participant number 1 – peer educator)

*“I think it will be received very positively. As I said, it’s a very bright manual. It’s very colourful, it’s got lots of pictures and when I looked at it, that was the first thing mostly that attracted me to it. It’s vibrant, it’s interesting… So, it wasn’t so boring for you to go and touch it, because sometimes you would find on pamphlets or manuals that the moment you look at it… I will make an example of our old peer educators’ manual. It was black and white, you know, sitting there, its thick, even if you want to go to it, it’s like okay, you just go to the page, okay no, I will do it some other time.”* (Participant number 2 – peer educator)

*“This one is very colourful, lots of pictures, and you can get the message from just looking at the pictures.”* (Participant number 3 – peer educator)

*“Things that we wanted outlined and changed and maybe, you know, instead of having this only word theory we would have for the handwashing, we would have these hands showing us with basins and all that, and I saw that all was implemented at the final manual.”* (Participant number 2 – peer educator)

*“I remember the first time we wanted a few words simplified, but this time around when I checked on it, there wasn’t a problem. Everything was understandable.”* (Participant number 3 – peer educator)

## Discussion

Health communication can play a vital role in health education interventions (47). This study used a community-based participatory research approach coinciding with health communication strategies to systemically develop, test, and implement a HIL and trainer’s manual on AMR. The high suitability scores of the leaflet (92% understandability, 97% actionability, 91% overall suitability) demonstrate that applying CBPR principles ensured the materials met the literacy and usability needs identified in the Introduction. The goal was to raise awareness on AMR amongst CHWs and peer educators by providing them with credible resources to further raise awareness and promote behaviour change within their communities (48). The purpose of developing and implementing health information material such as the HIL and trainer’s manual is to educate, inform and empower communities, which will further allow them to make better and knowledgeable decisions regarding their health (49, 50). Spreading awareness about the risks associated with AMR as well as its prevention and control is a global priority. Collaboration between researchers, stakeholders and the target group is an essential factor in developing effective and sustainable health interventions and is promoted across all research phases (51). The collaborative approach was successful in this study; from identifying the gaps and challenges on AMR to developing and testing the AMR HIL and trainer’s manual up until dissemination of the health information materials. This participatory approach received a positive response from the participants as well, as shown in their SSIs and FDGs. This empowerment was also reflected in the measurable outcomes of the workshops, where peer educators’ AMR knowledge increased, confirming that the intervention achieved its stated methodological goal of strengthening awareness through participatory design.

Social networks should be considered when it comes to understanding and influencing the behaviour of communities (52). The social network in Figure 4 illustrating the interactions between all stakeholders highlights the collaborative community-based approach used in this study. The postgraduate student, i.e., researcher had a two-way and full interaction with the academic staff/supervisors, Eastern Cape (Makana Sub-District) Department of Health, CHWs, peer educators and the Rhodes University Health Care Centre. The researcher was overseen by academic staff/supervisors, who thus had a two-way and full communication with the students. Based on building sustainable collaborative links and communication for more than a decade with the Eastern Cape (Makana Sub-District) Department of Health and with the Rhodes University Health Care Centre, and for over four years with peer educators and CHWs while implementing several research projects, the academic staff and/or supervisors had communication feedback loops, as and when required. The researcher communicated with the District Pharmacist from the Eastern Cape (Makana Sub-District) Department of Health with regards to organising FDGs with CHWs from seven PHC clinics. Upon the Eastern Cape (Makana Sub-District) Department of Health inviting the CHWs from each PHC clinic, the researcher then had a two-way and frequent interaction with the CHWs, particularly during the FDGs prior to HIL development and when obtaining feedback on the HIL drafts. The CHWs would then disseminate the HIL to all CHWs and amongst communities when going for home visits. The researcher communicated with the peer educators at Rhodes University and the Head Nurse at the Health Care Centre to organize workshops for the peer educators where the health information materials were developed and finalized, thus the researcher had a two-way and frequent interaction with the peer educators who were interlinked with the staff at the Rhodes University Health Care Centre, where the groups sometimes met for discussions. The peer educators would then disseminate the HIL and trainer’s manual to all Rhodes University support staff and amongst their communities.

Information design and the design process are key for effective health communication. The design process includes “user input, iteration and consideration of circumstances of use” (53). These aspects were successfully integrated in developing and testing the health information materials in this study. The intended users initially participated in the SSIs and FGDs to identify the gap prior to designing the HIL and then provided written feedback on the HIL drafts. Readability and suitability tests were reiterated on the first and final draft of the HIL, considering the low- to semi-literacy levels as well as the cultural aspects of the community. The finalised HIL is more likely to meet the desired outcomes and community’s needs if the target group is involved in the early development stages of the HIL (18, 54).

One of the key elements in HIL development is to determine the appropriateness of the HIL for the target population by quantitative and qualitative means (55). Between draft 1 and draft 3 of the HIL, the readability score improved from grade 16 to grade 14, thus making the third draft of the leaflet easier to read for the target population. Though the HIL was still classified as ‘difficult to read’, the feedback from the target population suggested that they understood the third draft and desired no more changes; thus, the readability score was maintained. A similar study was conducted with Rhodes University support staff in which a HIL titled ‘The Consequences of Alcohol Abuse’ resulted in an average readability score of grade 12 (46). It is important to note that the readability scores should not be the sole consideration when testing the HIL as readability formulas come with limitations – they rely on the length of a sentence and the number of syllables in a word (43). This means that a word may be easy to understand but if it consists of multiple syllables, it may be depicted as ‘difficult to read’ by readability formulas. The above is particularly common with medical terms such as “antibiotics,” “bacteria” and “vaccinations” which were used in this HIL. Additionally, readability formulas do not take formatting such as the font size and use of colour into account, which can affect the readability of information materials (50).

The challenge arose from the fact that final HIL was developed for the Grade 14 level of education. This is a problem as the target level was grade 10–12. During the development of the HIL, there was a decrease in the level of education that the individual drafts of the HIL were matching. The final draft of the HIL could not be changed or optimised any further, as the information content and usefulness in the AMR prevention/development management would be compromised. The level of education will need to be increased before the use of the HIL by providing additional training for the peer educators and/or CHWs. College or university level course on basic microbiology, infection control and additional AMR subject matter. This would provide a possible way to bridge the grade 14 and grade 10–12 intended gaps. The health promotion materials where first developed in English, as this is the first language of the healthcare and government in South Africa. At the same time, proper messaging has been highlighted as a problem in AMR combatting (56). Driving the development of more AMR materials in languages other than English is necessary. These should be developed based on the in-situ AMR experiences as was done in this study. A more broader testing of the health promotion materials developed here should be done for their applicability. The strategy used by the authors to engage the CHWs and peer educators are inclusive and go in line with the principles of communicative ecology.

The PEMAT and SAM are comprehensive instruments used to assess health information materials for aspects such as content, literacy, graphics, layout and typography, cultural appropriateness motivation, actionability and motivation (42–44). The SAM instrument categorises material as either superior (70–100%), adequate (40–69%) or not suitable (0–39%) (43). Between draft 1 and draft 3 of the HIL, the PEMAT understandability score improved from 87 to 92%, and the PEMAT actionability score improved from 92 to 97%. Between draft 1 and draft 3 of the HIL, the SAM score improved from 79 to 91%, proving that the HIL was suitable for the target population and of superior quality. Another study conducted with Rhodes University support staff that used the SAM instrument reported lower scores – 62.5% of the overall suitability of the HIL was rated as “superior”, 32.5% was rated as “adequate”, and 5% was rated as “not suitable”. The same study resulted in a similar PEMAT understandability score of 84.05% but a lower PEMAT actionability score of only 50% (46). Evidently, the HIL from the current study achieved higher SAM and PEMAT scores.

Whilst developing HILs, it is also important to consider communicative aspects such as the use of simplified language, and limited key messages with incentives that are action focussed and provide the community with clear instructions (75). In this case, the incentive is the appropriate use of antibiotics to reduce the emergence of AMR. Another study which evaluated PILs suggested that short (A4-sized page) and structured HILs with a legible font size (minimum point size of 12), relevant images and a clear message are more likely to be effective (18, 57). For HILs to be effective, they must be “noticed, read, understood, believed, and remembered” (50). Individuals with low literacy find the use of images alongside text especially useful to enhance usability, understandability and recollection of information materials (18, 58). It is important to note that for images to serve their purpose successfully they should be culturally appropriate for local communities (59). Most of the participants in this study expressed a positive response towards the use of images in the HIL, showing the importance of using graphics when it comes to health communication. The target group also emphasized on having the HIL presented in IsiXhosa, their home language. For this reason, the finalized HIL was then translated into IsiXhosa and disseminated amongst the CHWs and PAs from the seven PHC clinics to increase outreach, particularly to patients in the community.

Alongside good written materials, verbal communication plays an important role with regards to end-user satisfaction and adherence. Good communication skills include the use of plain and simple language, talking at a steady pace and not too fast, repeating key messages, and having a collaborative approach with the users where their preferences and needs are taken into consideration (60, 61). In this study, all workshops and discussions were conducted in a similar manner. The researcher verbally reviewed the Participant Invitation Letter as well as the Participant Informed Consent Form with the participants to confirm understandability prior to conducting the workshops and discussions. Moreover, an IsiXhosa interpreter was present at the first peer educators’ workshop to further rule out communication difficulties and the language barrier for participants with low- to semi-literacy levels, which further ensured communication in a linguistically and culturally appropriate manner. However, another South African study showed that the use of interpreters increased the risk of medical mistakes (62), and according to another study conducted in the USA, using *ad hoc* interpretation was shown to worsen communication than not using any interpretation at all (63).

To further increase outreach, peer educators from Rhodes University were included in the study and a mandatory workshop was conducted for all the peer educators, part of which, the topic of AMR was introduced to them. The intended users (*N* = 21) participated in the first workshop to increase their awareness on AMR, and the pre- and post-workshop questionnaires were used to quantify the above. The pre-workshop questionnaire received an average score of 55.84% as compared to the post-workshop questionnaire which received an average of 73.59%, indicating that the information was easy to be introduced and obtained in limited time, and that the participants’ knowledge on AMR increased significantly after the workshop was conducted. It is important to note that before the workshop, 90.48% of the participants believed that antibiotics could help to fight the flu. This result significantly dropped down to 42.86% after the workshop, indicating clarity on an important contributing factor to the emergence of AMR. The pre- and post-workshop scores were tested for the statistically significant difference using the Past 3.0–5.0 statistical software package (see https://www.nhm.uio.no/english/research/resources/past/ for details, website accessed on 19th July 2025). There was a statistically significant difference in the questionnaire scores with the respective (*p*-value < 0.0100). A similar AMR survey showed that 60.6% of adults between the age group of 35–44 years and 47.6% of adults between the age group of 45–54 years wrongly believed common colds and flu can be treated with antibiotics (64). In addition, 64% of the respondents across the 12 countries surveyed by WHO also mistakenly believed the same statement (65). The participants in the current study appreciated the AMR workshop and indicated that they learnt a lot, and will share the information with others, including their family and friends. One participant stated, “I learn[t] a lot because I use[d] to share with my families and leave [the medicine], when I feel sick eat [the medicine] again” which further highlighted two key contributing factors to the emergence of AMR – not completing the treatment course and sharing antibiotics.

The intended users (*N* = 7) participated in the second workshop to go through the AMR HIL to further increase their knowledge on AMR and to reiterate the key points from the first workshop, and the pre- and post-workshop questionnaires were used to quantify the above. The pre-workshop questionnaire received an average score of 58.82% as compared to the post-workshop questionnaire which received an average of 63.87%, indicating that the participants’ knowledge on AMR probably increased after the workshop was conducted. It was observed that the average scores of the pre- and post-workshop questionnaire of the second workshop were lower as compared to those of the first workshop, suggesting that retention of knowledge reduced over time. These scores were also not statistically significantly different (Mann–Whitney test at 5% level of significance, *p*-value > 0.0600). Similarly, a study explored retention of knowledge amongst teachers who were trained to teach sign language to children with disabilities during a one-day workshop, which demonstrated that though participants were able to pick up language signals, sign knowledge decreased after six weeks and 12 weeks (66). In contrast to both studies, another study found that participant knowledge significantly improved and retained for up to 12 weeks after the one-day AMR workshop (67). However, Ahmed’s study was conducted amongst students between the ages of 14–16 years (67), thus different demographics and study topics can result in different outcomes. For the second workshop, the results for question four of the questionnaire indicated that the question was misunderstood as the participants indicated “Yes” or “No” for each statement alone, i.e., whether each statement is true or false on its own without referring to the actual question being asked. It was observed that Yes/No and multiple-choice questions can sometimes be a challenge for participants with low- to semi-literacy levels as health literacy comprises of a wide range of skills, including reading and writing (61). This went on to show that the participants understood the content that was discussed at the workshop and were able to respond verbally, however this did not fully reflect in their written responses. For this reason, this study used qualitative and quantitative methods to assess the level of understanding amongst the target group. Like in the previous workshop, participants showed a significant increase in knowledge regarding the use of antibiotics for a flu and/or cold. Before the workshop, 71.43% of the participants believed that one should get antibiotics from the clinic as soon as they get the flu or cold. This result significantly dropped down to 14.29% after the workshop, indicating that an important factor was learnt. The participants appreciated the workshop and indicated that they learnt a lot, and will share the information with others, including their family and friends. One participant stated, “I like to be part of this because [this] is where I increase my knowledge and understanding of antibiotics” and another participant stated, “We have learnt a lot about antibiotics and can be helpful to our families and friends.” The importance of health communication was further highlighted when one participant stated, “Yes, the clinics must tell us if you do not finish your medicine, you will not get better at all.” Due to the shortage of health workforce and resources overburdening the healthcare systems in South Africa (68), many healthcare professionals do not have sufficient time for patient education and counselling (69). Therefore, the dissemination of health information materials developed particularly for individuals with low- to semi-health literacy levels will assist in raising awareness and allowing users to make better and more informed health-related decisions (70).

The format of the HIL and trainer’s manual were highly appreciated by the peer educators. When targeting users with low literacy, it is vital to use plain and simple language as far as possible as it increases the readability and understandability. This includes using the active voice; using short and simple sentences with common words; limiting the use of medical jargon; defining medical and/or technical terms when required; providing information in sections with appropriate headings and sub-headings; using bullet points where feasible; using at least a 12-point font size; and spacing out information (60, 61). Formatting components such as uppercase, italics, and abbreviations should be avoided as users with low literacy levels may find them difficult to understand (60, 61, 71). The written information should be complemented with an adequate number of context-specific graphics along with captions to convey the information more clearly (60, 71, 72). To promote behaviour change amongst the target group, health information materials should be actionable by clearly stating what steps should be taken towards achieving the goal (60, 72). These key factors were taken into consideration during this study as the medical terms were defined, the impact of AMR was explained, the main points were emphasized, and the actions to be taken by the target group were clearly listed.

The second draft of the trainer’s manual, which was presented in both English and IsiXhosa, was very well received by the peer educators. This phase also allowed for the IsiXhosa version of the trainer’s manual to be pilot tested to confirm its accuracy and understandability amongst its users, in this case being peer educators. It is important for multilingual written materials to be professionally translated and then tested in the field prior to dissemination (60). The language barrier in the healthcare system poses major challenges in providing quality healthcare, patient satisfaction as well as adequate communication. Communities with limited English proficiency are not only more likely to misunderstand the information presented to them but are also more likely to lack adherence (73). It is therefore important to translate health information materials into the native languages, in this case being IsiXhosa and Afrikaans.

The peer educators found the trainer’s manual to be an extremely useful resource in the context of the COVID-19 pandemic as the manual provided detailed infographics on hand hygiene and the importance of frequent hand washing, as well as the differentiation between bacteria and viruses with regards to the use of antibiotics. COVID-19 is increasingly becoming a contributing factor to AMR due to the increased use of antibiotics for COVID-19 patients (74). The trainer’s manual therefore allowed the users to better understand the dos and don’ts during the pandemic due to the knowledge previously gained.

The trainers manual and the HIL, which had been developed in this study, are locally focused on the AMR situation in Makana Local Municipality. At the same time, they are based on the global and general knowledge about factors that often control or contribute to the development of AMR in a specific location [see Sharma et al. (23) for more information]. Makana Local Municipality is a multilingual area with at least three languages being spoken. Cultural and socio-economic factors will, in conjunction with the language barriers and potential lack of training of formal healthcare workers and CHWs, impact the success of the health promotion campaigns to tackle AMR (23). In this context, the current study used an inclusive and multi-stakeholder approach to design the health promotion materials and also to engage with CHWs in the development of their knowledge about AMR. The healthcare professionals who engaged in the development of the HIL and the trainer’s manual were engaged as voices of the local cultural and social fabric of society in Makana. As a result, the developed approach provides a viable option to tackle AMR and address any potential cultural and social/language barriers to the success of the endeavour. By explicitly showing that materials classified as ‘difficult’ by formula-based readability scores were nonetheless understood and retained in practice, the findings close the loop between methodological evaluation tools and the study’s broader objective of creating context-specific, sustainable communication resources.

### Limitations of the study

The approach to tackling AMR in Makana Local Municipality was based here on the training and co-creation of the health promotion materials with the peer educators and CHWs from the Makana area. The workshop or training intervention had some effect on the knowledge of the CHWs and peer educators about AMR, but more knowledge must be developed and run with the CHWs, peer educators and also formal healthcare workers in the Makana Local Municipality. The trainer’s manual and the HIL must be tested in a wider context. The current questionnaire, the study results and discussion provide a basis for locally-centred health promotion to tackle AMR. Limitations of the current study are the need for further education of peer educators and the CHWs. The developed materials must still therefore be further tested at a wider scale and over an extended period of time in Makana Local Municipality. Discussion and the qualitative feedback can be exploited to drive such wider testing of the HIL and trainer’s manual.

## Conclusion

This study highlighted the importance of information design for effective health communication, particularly for AMR. The AMR HIL scored a final readability of grade 14. Though the readability tests classified the HIL as “difficult to read”, the feedback from all stakeholders suggested that the HIL was suitable for them and required no further changes. The high PEMAT and SAM scores proved that the HIL was understandable, actionable, suitable, and culturally appropriate for the target population. The AMR trainer’s manual was very well received by the target group, and the workshops proved to increase awareness on AMR amongst peer educators at Rhodes University. Information design is vital for effective health communication, which is a key strategy to tackle and prevent AMR. By involving the target group in the development process, the health information materials are likely to be more effective and sustainable. The participatory approach of this study not only empowers CHWs and peer educators but provides them with a handy resource for future use. These participatory programmes empower CHWs and peer educators to address the communication gaps between healthcare professionals and the community through health communication interventions.

## Data Availability

The original contributions presented in the study are included in the article/Supplementary material, and further inquiries can be directed to the corresponding author.

## References

[1] WHO. (2015). Global action plan on antimicrobial resistance. WHO. Available online at: https://www.who.int/publications/i/item/9789241509763

[2] Charoenboon N Haenssgen MJ Warapikuptanun P Xayavong T Khine Zaw Y. Translating antimicrobial resistance: a case study of context and consequences of antibiotic-related communication in three northern Thai villages. Palgrave Commun. (2019) 5. doi: 10.1057/s41599-019-0226-9

[3] Haenssgen MJ Charoenboon N Zanello G Mayxay M Reed-Tsochas F Lubell Y. Antibiotic knowledge, attitudes and practices: new insights from cross-sectional rural health behaviour surveys in low-income and middle-income South-East Asia. BMJ Open. (2019) 9:e028224. doi: 10.1136/bmjopen-2018-028224, PMID: 31434769 PMC6707701

[4] Haenssgen MJ Xayavong T Charoenboon N Warapikuptanun P Khine Zaw Y. The consequences of AMR education and awareness raising: outputs, outcomes, and Behavioural impacts of an antibiotic-related educational activity in Lao PDR. Antibiotics. (2018) 7:95. doi: 10.3390/antibiotics7040095, PMID: 30388824 PMC6316454

[5] Huttner B Saam M Moja L Mah K Sprenger M Harbarth S. How to improve antibiotic awareness campaigns: findings of a WHO global survey. BMJ Glob Health. (2019) 4:e001239. doi: 10.1136/bmjgh-2018-001239, PMID: 31179029 PMC6528771

[6] Price L Gozdzielewska L Young M Smith F MacDonald J McParland J. Effectiveness of interventions to improve the public’s antimicrobial resistance awareness and behaviours associated with prudent use of antimicrobials: a systematic review. J Antimicrob Chemother. (2018) 73:1464–78. doi: 10.1093/jac/dky076, PMID: 29554263

[7] WHO (2018). Strategic and technical advisory group on antimicrobial resistance (STAG-AMR)—Report of ninth meeting 26–27 February 2018 WHO headquarters, Geneva. Available online at: https://www.who.int/groups/strategic-and-technical-advisory-group-on-antimicrobial-resistance

[8] O’Neill J. (2014). Antimicrobial resistance: Tackling a crisis for the health and wealth of nations—The review on antimicrobial resistance. Available online at: https://amr-review.org/sites/default/files/AMR%20Review%20Paper%20-%20Tackling%20a%20crisis%20for%20the%20health%20and%20wealth%20of%20nations_1.pdf

[9] Wutzke SE Artist MA Kehoe LA Fletcher M Mackson JM Weekes LM. Evaluation of a national programme to reduce inappropriate use of antibiotics for upper respiratory tract infections: effects on consumer awareness, beliefs, attitudes and behaviour in Australia. Health Promot Int. (2007) 22:53–64. doi: 10.1093/heapro/dal034, PMID: 17046966

[10] West LM Cordina M. Educational intervention to enhance adherence to short-term use of antibiotics. Res Soc Adm Pharm. (2019) 15:193–201. doi: 10.1016/j.sapharm.2018.04.011, PMID: 29685459

[11] de Bont EGPM Alink M Falkenberg FCJ Dinant G-J Cals JWL. Patient information leaflets to reduce antibiotic use and reconsultation rates in general practice: a systematic review. BMJ Open. (2015) 5:e007612. doi: 10.1136/bmjopen-2015-007612, PMID: 26041493 PMC4458684

[12] Colledge A Car J Donnelly A Majeed A. Health information for patients: time to look beyond patient information leaflets. J R Soc Med. (2008) 101:447–53. doi: 10.1258/jrsm.2008.080149, PMID: 18779246 PMC2587380

[13] Kotwani A Wattal C Katewa S Joshi PC Holloway K. Factors influencing primary care physicians to prescribe antibiotics in Delhi India. Fam Pract. (2010) 27:684–90. doi: 10.1093/fampra/cmq059, PMID: 20660529

[14] Basch CH Ethan D MacLean SA Fera J Garcia P Basch CE. Readability of prostate Cancer information online: a cross-sectional study. Am J Mens Health. (2018) 12:1665–9. doi: 10.1177/1557988318780864, PMID: 29888641 PMC6142125

[15] Hadjipavlou M Khan S Rane A. Readability of patient information leaflets for urological conditions and treatments. J Clin Urol. (2013) 6:302–5. doi: 10.1177/2051415813489554

[16] Badarudeen S Sabharwal S. Assessing readability of patient education materials: current role in Orthopaedics. Clin Orthop Relat Res. (2010) 468:2572–80. doi: 10.1007/s11999-010-1380-y, PMID: 20496023 PMC3049622

[17] Høstgaard AM Bertelsen P Nøhr C. Methods to identify, study and understand end-user participation in HIT development. BMC Med Inform Decis Mak. (2011) 11:57. doi: 10.1186/1472-6947-11-57, PMID: 21955493 PMC3196903

[18] Van Beusekom MM Grootens-Wiegers P Bos MJW Guchelaar H-J van den Broek JM. Low literacy and written drug information: information-seeking, leaflet evaluation and preferences, and roles for images. Int J Clin Pharm. (2016) 38:1372–9. doi: 10.1007/s11096-016-0376-4, PMID: 27655308 PMC5124048

[19] Llor C Bjerrum L. Antimicrobial resistance: risk associated with antibiotic overuse and initiatives to reduce the problem. Therap Adv Drug Safety. (2014) 5:229–41. doi: 10.1177/2042098614554919, PMID: 25436105 PMC4232501

[20] Siegfried N Draper B Draper G Porter M Bonaconsa C Hunter J. A contextualisation approach to health promotion guideline development in South Africa. S Afr Med J. (2018) 108:1036–41. doi: 10.7196/SAMJ.2018.v108i12.13129, PMID: 30606288

[21] Onya H. Health promotion in South Africa. Promot Educ. (2007) 14:233–7. doi: 10.1177/10253823070140041001, PMID: 18372875

[22] Perez AM Ayo-Yusuf OA Hofman K Kalideen S Maker A Mokonoto D. Establishing a health promotion and development foundation in South Africa. S Afr Med J. (2013) 103:147–9. doi: 10.7196/SAMJ.6281, PMID: 23472686

[23] Sharma S Srinivas CS Tandlich R. Communicative ecology and antimicrobial resistance management in the primary healthcare clinics in an eastern cape municipality in South Africa: basis for health promotion and tack-ling the problem. Front Public Health. (2025) manuscript number 1668313

[24] Luhmann N. What is communication? Commun Theory. (1992) 2:251–9. doi: 10.1111/j.1468-2885.1992.tb00042.x

[25] Redfern J Bowater L Coulthwaite L Verran J. Raising awareness of antimicrobial resistance among the general public in the UK: the role of public engagement activities. JAC Antimicrob Resist. (2020) 2:dlaa012. doi: 10.1093/jacamr/dlaa012, PMID: 34222970 PMC8210175

[26] WHO (2019). Turning plans into actions for antimicrobial resistance (AMR)—Working paper 2.0: Implementation and coordination Available online at: https://apps.who.int/iris/bitstream/handle/10665/311386/WHO-WSI-AMR-2019.2-eng.pdf

[27] The Commonwealth Fund (2016). Brazil’s family health strategy: using community health workers to provide primary care. Available online at: https://www.commonwealthfund.org/sites/default/files/documents/___media_files_publications_case_study_2016_dec_1914_wadge_brazil_family_hlt_strategy_frugal_case_study_v2.pdf

[28] Tulenko K Mogedal S Afzal MM Frymus D Oshin A Pate M. Community health workers for universal health-care coverage: from fragmentation to synergy. Bull World Health Organ. (2013) 91:847–52. doi: 10.2471/BLT.13.118745, PMID: 24347709 PMC3853952

[29] Olaniran A Smith H Unkels R Bar-Zeev S van der Broek N. Who is a community health worker? A systematic review of definitions. Glob Health Action. (2017) 10:1272223. doi: 10.1080/16549716.2017.1272223, PMID: 28222653 PMC5328349

[30] Ozano K Simkhada P Thann K Khatri R. Improving local health through community health workers in Cambodia: challenges and solutions. Hum Resour Health. (2018) 16:2. doi: 10.1186/s12960-017-0262-8, PMID: 29304869 PMC5756401

[31] Abdi F Simbar M. The peer education approach in adolescents-narrative review article. Iran J Public Health. (2013) 42:1200–6. 26171331 PMC4499060

[32] Demirezen D Karaca A Konuk Sener D Ankarali H. Agents of change: the role of the peer education program in preventing adolescent substance abuse. J Child Adolesc Subst Abuse. (2019) 28:376–87. doi: 10.1080/1067828X.2020.1766618, PMID: 8918852

[33] Frantz JM. A peer-led approach to promoting health education in schools: the views of peers. S Afr J Educ. (2015) 35:01–7. doi: 10.15700/201503070006

[34] Kannappan S Shanmugam K. Peer educators as change leaders – effectiveness of peer education process in creating awareness on reproductive health among women Workers in Textile Industry. Ind J Commun Med. (2019) 44:252–5. doi: 10.4103/ijcm.IJCM_6_19, PMID: 31602114 PMC6776950

[35] Winterbauer NL Bekemeier B VanRaemdonck L Hoover AG. Applying community-based participatory research partnership principles to public health practice-based research networks. SAGE Open. (2016). 6:10.1177/2158244016679211. doi: 10.1177/2158244016679211 PMC6533003 31131152

[36] Makana Local Municipality (2017-2022). Final Makana municipality integrated development plan 2017–2022. Available online at: https://www.makana.gov.za/wp-content/uploads/2013/06/IDP-2017-2022-Final.pdf (website accessed on 14th July 2025).

[37] Nhokodi T. Nqowana T. Dube C. S. Tandlich R. (2017). Identification of bacteria in rainwater samples from South Africa. Published in the peer-reviewed proceedings from the 9th air and water components of the environment conference be held in Cluj-Napoca, Romania from 17th until 19th march 2017, pp. 333-337 (ISSN: 2067-743)

[38] Sharma S Tandlich R Docrat M Srinivas S. Antibiotic procurement and ABC analysis for a comprehensive primary health care clinic in the eastern cape province, South Africa. South Afr J Infect Dis. (2020) 35:134. doi: 10.4102/sajid.v35i1.134, PMID: 34604377 PMC8477259

[39] Iheanetu C Tandlich R. Water provision under the COVID-19 lockdown conditions: snapshot of microbial quality of alternative sources, the associated costs and carbon footprints. Vedelem Tudomany (Protection Science). (2022) VII:162–90.

[40] Dowse R Okeyo S Sikhondze S Khumalo N. Pharmaceutical indication pictograms for low literacy viewers: health literacy and comprehension. Health SA. (2023) 28:2192. doi: 10.4102/hsag.v28i0.2192, PMID: 37927939 PMC10623492

[41] Automatic Readability Checker—A free text readability consensus calculator. (2022). Readabilityformulas.Com. Available online at: https://www.readabilityformulas.com/free-readability-formula-tests.php

[42] Shoemaker S. J. Wolf M. S. Brach C. (2013). The patient education materials assessment tool (PEMAT) and user’s guide. Available online at: https://www.ahrq.gov/sites/default/files/publications2/files/pemat_guide_0.pdf 10.1016/j.pec.2014.05.027 PMC5085258 24973195

[43] Jahan S Al-Saigul AM Alharbi AM Abdelgadir MH. Suitability assessment of health education brochures in Qassim province, Kingdom of Saudi Arabia. J Fam Community Med. (2014) 21:186–92. doi: 10.4103/2230-8229.142974, PMID: 25374471 PMC4214009

[44] Suitability Assessment of Materials (SAM) for evaluation of health-related information for adults. (2008). Available online at: https://ogg.osu.edu/media/documents/health_lit/HOSAM2006.pdf...after the first link

[45] Manhanzva R. I. (2019). Antimicrobial resistance awareness program at settlers hospital. Master of pharmacy practice thesis, Rhodes University. Available online at: http://hdl.handle.net/10962/97712

[46] Sharma S Marara P Townsend N Srinivas S. Developing and testing a culturally sensitive health information leaflet on the consequences of alcohol abuse. Ethiop J Health Dev. (2018) 32:46–53. doi: 10.4314/ejhd.v32i1

[47] Zhao X. Health communication campaigns: a brief introduction and call for dialogue. Int J Nurs Sci. (2020) 7:S11–5. doi: 10.1016/j.ijnss.2020.04.009, PMID: 32995373 PMC7501494

[48] Smith M Mateo KF Morita H Hutchinson C Cohall AT. Effectiveness of a multifaceted community-based promotion strategy on use of GetHealthyHarlem.org, a local community health education website. Health Promot Pract. (2015) 16:480–91. doi: 10.1177/1524839915571632, PMID: 25695422

[49] Asbury N Walshe A. Involving women with breast cancer in the development of a patient information leaflet for anticipatory nausea and vomiting. Eur J Oncol Nurs. (2005) 9:33–43. doi: 10.1016/j.ejon.2004.07.003, PMID: 15774339

[50] Protheroe J Estacio EV Saidy-Khan S. Patient information materials in general practices and promotion of health literacy: an observational study of their effectiveness. Br J Gen Pract. (2015) 65:e192–7. doi: 10.3399/bjgp15X684013, PMID: 25733441 PMC4337308

[51] Morton KL Atkin AJ Corder K Suhrcke M Turner D van Sluijs EMF. Engaging stakeholders and target groups in prioritising a public health intervention: the creating active school environments (CASE) online Delphi study. BMJ Open. (2017) 7:e013340. doi: 10.1136/bmjopen-2016-013340, PMID: 28087549 PMC5253605

[52] Ellis J Vassilev I Kennedy A Moore M Rogers A. Help seeking for antibiotics; is the influence of a personal social network relevant? BMC Fam Pract. (2019) 20:63. doi: 10.1186/s12875-019-0955-2, PMID: 31088394 PMC6518744

[53] Walker S. Effective antimicrobial resistance communication: the role of information design. Palgrave Commun. (2019) 5:24. doi: 10.1057/s41599-019-0231-z

[54] Rajesh R Vidyasagar S Varma M Sharma S. Design and evaluation of pictograms for communicating information about adverse drug reactions to antiretroviral therapy in Indian human immunodeficiency virus positive patients. J Pharmac Biomed Sci. (2012) 16

[55] Goldsmith MR Bankhead CR Austoker J. Synthesising quantitative and qualitative research in evidence-based patient information. J Epidemiol Community Health. (2007) 61:262–70. doi: 10.1136/jech.2006.046110, PMID: 17325406 PMC2652927

[56] Mendelson M Balasegaram M Jinks T Pulcini C Sharland M. Antibiotic resistance has a language problem. Nature. (2017) 545:23–5. doi: 10.1038/545023a, PMID: 28470219

[57] Liu F Abdul-Hussain S Mahboob S Rai V Kostrzewski A. How useful are medication patient information leaflets to older adults? A content, readability and layout analysis. Int J Clin Pharm. (2014) 36:827–34. doi: 10.1007/s11096-014-9973-2, PMID: 24986267

[58] Mansoor LE Dowse R. Effect of pictograms on readability of patient information materials. Ann Pharmacother. (2003) 37:1003–9. doi: 10.1345/aph.1C449, PMID: 12841808

[59] Barros IM Alcântara TS Mesquita AR Bispo ML Rocha CE Moreira VP. Understanding of pictograms from the United States Pharmacopeia dispensing information (USP-DI) among elderly Brazilians. Patient Prefer Adherence. (2014) 8:1493–501. doi: 10.2147/PPA.S65301, PMID: 25378914 PMC4219639

[60] Garcia SF Hahn EA Jacobs EA. Addressing low literacy and health literacy in clinical oncology practice. J Support Oncol. (2010) 8:64–9. 20464884 PMC3127453

[61] Hersh L Salzman B Snyderman D. Health literacy in primary care practice. Am Fam Physician. (2015) 92:118–24. 26176370

[62] Hunter-Adams J Rother H-A. A qualitative study of language barriers between south African health care providers and cross-border migrants. BMC Health Serv Res. (2017) 17:97. doi: 10.1186/s12913-017-2042-5, PMID: 28143514 PMC5282629

[63] Flores G Abreu M Barone CP Bachur R Lin H. Errors of medical interpretation and their potential clinical consequences: a comparison of professional versus ad hoc versus no interpreters. Ann Emerg Med. (2012) 60:545–53. doi: 10.1016/j.annemergmed.2012.01.025, PMID: 22424655

[64] Hong Kong Department of Health. (2018). General public’s knowledge, attitude and practice survey on antimicrobial resistance 2016/17. Available online at: https://www.chp.gov.hk/files/pdf/kap_on_amr_main_report.pdf

[65] WHO (2015). Antibiotic resistance: Multi-country public awareness survey. Available online at: https://www.who.int/news/item/16-11-2015-who-multi-country-survey-reveals-widespread-public-misunderstanding-about-antibiotic-resistance 10.3390/antibiotics14060599 PMC12189553 40558189

[66] Smidt A Markoulli C Wine C Chang E Turnbull H Huzmeli A. Retention of signs following a one-day key word sign training. Br J Learn Disabil. (2019) 47:50–8. doi: 10.1111/bld.12257

[67] Ahmed R Bashir A Brown JEP Cox JAG Hilton AC Jordan SL. Aston University’s antimicrobial resistance (AMR) roadshow: raising awareness and embedding knowledge of AMR in key stage 4 learners. Infect Prev Pract. (2020) 2:100060. doi: 10.1016/j.infpip.2020.100060, PMID: 34368704 PMC8336141

[68] Maphumulo WT Bhengu BR. Challenges of quality improvement in the healthcare of South Africa post-apartheid: a critical review. Curationis. (2019) 42:a1901. doi: 10.4102/curationis.v42i1.1901, PMID: 31170800 PMC6556866

[69] Paterick TE Patel N Tajik AJ Chandrasekaran K. Improving health outcomes through patient education and partnerships with patients. Proc (Baylor Univ Med Cent). (2017) 30:112–3. doi: 10.1080/08998280.2017.11929552, PMID: 28152110 PMC5242136

[70] Patel A Bakina D Kirk J von Lutcken S Donnelly T Stone W. Patient counseling materials: the effect of patient health literacy on the comprehension of printed prescription drug information. Res Soc Adm Pharm. (2018) 14:851–62. doi: 10.1016/j.sapharm.2018.04.035, PMID: 29887494

[71] CDC (2009) Simply put—A guide for creating easy-to-understand materials. Available online at: https://stacks.cdc.gov/view/cdc/11938

[72] WHO (2017) WHO strategic communications framework for effective communications. Available online at: https://www.who.int/docs/default-source/documents/communicating-for-health/communication-framework.pdf

[73] Al Shamsi H Almutairi AG Al Mashrafi S Al Kalbani T. Implications of language barriers for healthcare: a systematic review. Oman Med J. (2020) 35:e122. doi: 10.5001/omj.2020.40, PMID: 32411417 PMC7201401

[74] Pelfrene E Botgros R Cavaleri M. Antimicrobial multidrug resistance in the era of COVID-19: a forgotten plight? Antimicrob Resist Infect Control. (2021) 10:21. doi: 10.1186/s13756-021-00893-z, PMID: 33514424 PMC7844805

[75] Warde F Papadakos J Papadakos T Rodin D Salhia M Giuliani M. Plain language communication as a priority competency for medical professionals in a globalized world. Can Med Educ J. (2018) 9e52–e59., PMID: 30018684 PMC6044302

