# Supplementary material for: Development of health information materials on antimicrobial resistance with lay workers in Grahamstown/Makhanda, South Africa
Source: Front Public Health. 2025 Oct 29;13:1542448. doi: 10.3389/fpubh.2025.1542448 (PMC12608051; doi:10.3389/fpubh.2025.1542448)
Supplement: Supplementary file 1 [file Data_Sheet_1.docx]

**Appendix I: AMR HIL Final Draft**


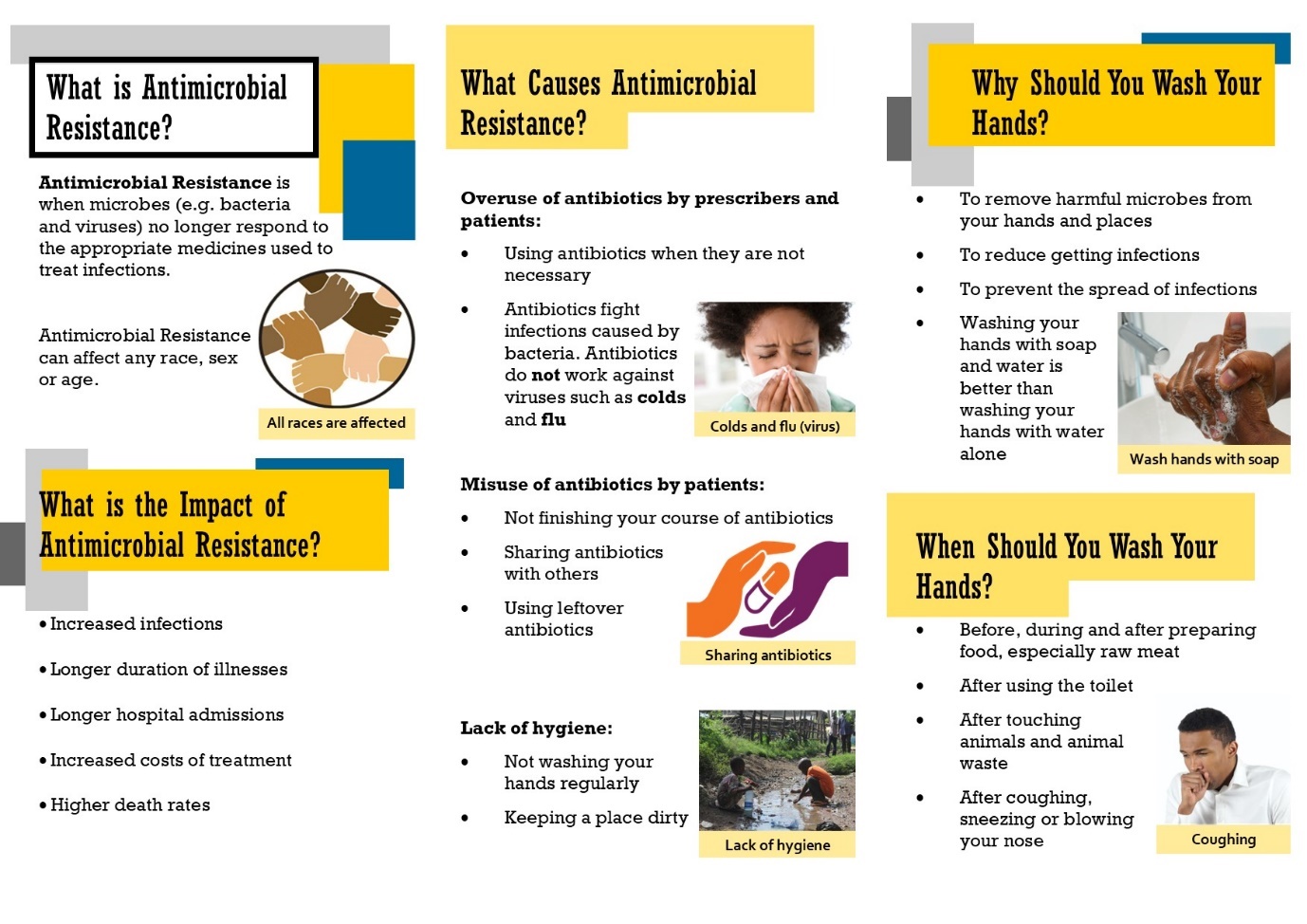


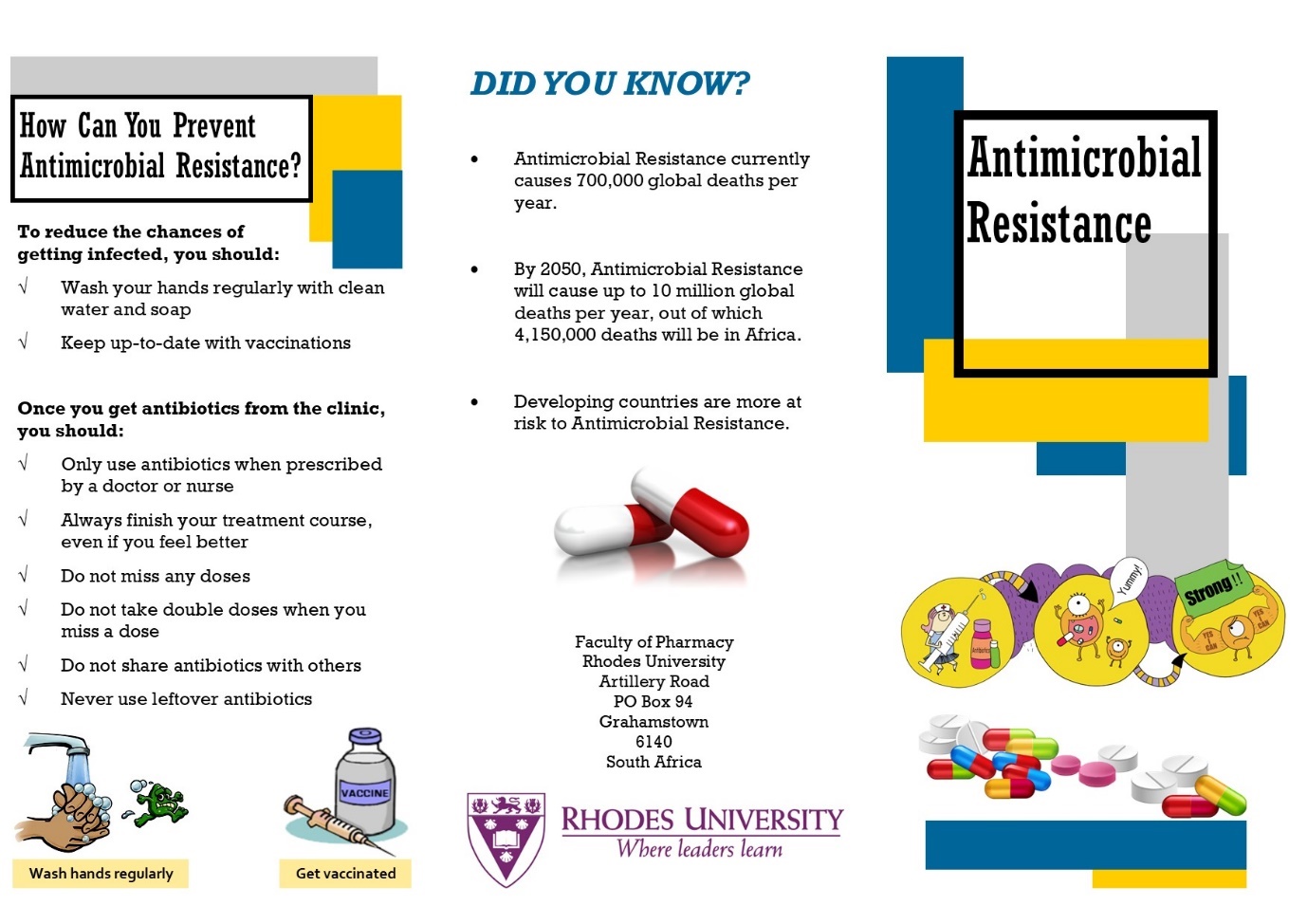


## **Patient Education Materials Assessment Tool (PEMAT) Template**

| **Patient Education Materials Assessment Tool for Printable Materials** | | | |
| --- | --- | --- | --- |
| Reviewer name:  Title of publication:  Date of review: | | | |
| **UNDERSTANDABILITY** | | | |
|  | Item | Response Option | Rating |
| **Topic: Content** | | | |
| 1. | The material makes its purpose completely evident. | Disagree=0, Agree=1 |  |
| 2. | The material does not include information or content that distracts from its purpose. | Disagree=0, Agree=1 |  |
| **Topic: Word Choice & Style** | | | |
| 3. | The material uses common, everyday language. | Disagree=0, Agree=1 |  |
| 4. | Medical terms are used only to familiarize audience with the terms. When used, medical terms are defined. | Disagree=0, Agree=1 |  |
| 5. | The material uses the active voice | Disagree=0, Agree=1 |  |
| **Topic: Use of Numbers** | | | |
| 6. | Numbers appearing in the material are clear and easy to understand. | Disagree=0, Agree=1, No numbers=N/A |  |
| 7. | The material does not expect the user to perform calculations. | Disagree=0, Agree=1, No numbers=N/A |  |
| **Topic: Organization** | | | |
| 8. | The material breaks or “chunks” information into short sections | Disagree=0, Agree=1,  Very short material*=N/A |  |
| 9. | The material’s sections have informative headers. | Disagree=0, Agree=1, Very short material*=N/A |  |
| 10. | The material presents information in a logical sequence. | Disagree=0, Agree=1 |  |
| 11. | The material provides a summary. | Disagree=0, Agree=1,  Very short material*=N/A |  |
| **Topic: Layout & Design** | | | |
| 12. | The material uses visual cues (e.g., arrows, boxes, bullets, bold, larger font, highlighting) to draw attention to key points. | Disagree=0, Agree=1  Video=N/A |  |
| **Topic: Use of Visual Aids** | | | |
| 13. | The material uses visual aids whenever they could make content more easily understood (e.g. illustration of healthy portion size) | Disagree=0, Agree=1 |  |
| 14. | The material’s visual aids reinforce rather than distract from the content | Disagree=0, Agree=1,  No visual aids=N/A |  |
| 15. | The material’s visual aids have clear titles or captions. | Disagree=0, Agree=1,  No visual aids=N/A |  |
| 16. | The material uses illustrations and photographs that are clear and uncluttered. | Disagree=0, Agree=1,  No visual aids=N/A |  |
| 17. | The material uses simple tables with short and clear row and column headings | Disagree=0, Agree=1,  No tables=N/A |  |

**Total Points: ____________________**

**Total Possible Points: ____________________**

**Understandability Score (%): ____________________**

**(Total Points / Total Possible Points) X 100**

| **ACTIONABILITY** | | | |
| --- | --- | --- | --- |
|  | Item | Response Option | Rating |
| 18. | The material clearly identifies at least one action the user can take. | Disagree=0, Agree=1 |  |
| 19. | The material addresses the user directly when describing actions. | Disagree=0, Agree=1 |  |
| 20. | The material breaks down any action into manageable, explicit steps. | Disagree=0, Agree=1 |  |
| 21. | The material provides a tangible tool (e.g., menu planners, checklists) whenever it could help the user take action | Disagree=0, Agree=1 |  |
| 22. | The material provides simple instructions or examples of how to perform calculations. | Disagree=0, Agree=1,  No calculations=NA |  |
| 23. | The material explains how to use the charts, graphs, tables, or diagrams to take actions. | Disagree=0, Agree=1,  No charts, graphs,  tables, or  diagrams=N/A |  |
| 24. | The material uses visual aids whenever they could make it easier to act on the instructions. | Disagree=0, Agree=1 |  |

**Total Points: ____________________**

**Total Possible Points: ____________________**

**Actionability Score (%): ____________________**

**(Total Points / Total Possible Points) X100**

* **A very short print material is defined as a material with two or fewer paragraphs and no more than 1 page in length.**

## **Suitability Assessment of Material (SAM) Template**

| **Suitability Assessment of Materials (SAM)** | | | |
| --- | --- | --- | --- |
| Name of reviewer |  | | |
| Title of publication |  | | |
| Date of review |  | | |
| Response Option: Superior=2, Adequate=1, Not suitable=0 | | | |
| 1.Content | | (a) Purpose is evident |  |
|  |  | (b)Content about behaviours |  |
|  |  | (c)Scope is limited |  |
|  |  | (d)Summary included |  |
| 2.Literacy demand | | (a)Reading grade level   - Superior= 5th grade or lower - Adequate=6th -8th grade - Not suitable= 9th grade and above |  |
|  |  | (b)Writing Style, active voice used |  |
|  |  | (c)Vocabulary |  |
|  |  | (d)Context given |  |
|  |  | (e)Advance organisers |  |
| 3.Graphics | | (a)Cover graphic shows purpose |  |
|  |  | (b)Type of graphics used |  |
|  |  | (c)Relevance of illustrations |  |
|  |  | (d)Lists and tables explained |  |
|  |  | (e)Captions used for graphics |  |
| 4.Layout and typography | | (a)Layout factors |  |
|  |  | (b)Typography |  |
|  |  | (c)Subheadings used |  |
| 5.Learning simulation and motivation | | (a)Interaction used |  |
|  |  | (b)Behaviours are modelled and specific |  |
|  |  | (c)Motivation |  |
|  | | **Total score** |  |

## **Brief Survey for AMR HIL Pilot Testing**

Participant Number..........................................

Age..........................................

Gender..........................................

Home Language..........................................

Profession....................................................................................

Name of Clinic....................................................................................

Date..........................................

**Pictogram Assessment**

1. Please indicate your answer by ticking under “Yes” or “No”, and comment to explain what each picture shows.

| **Picture 1**  **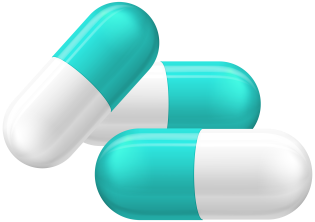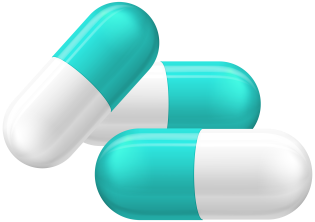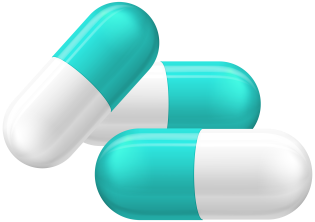** | **Do you understand the picture?** | | **Comment** |
| --- | --- | --- | --- |
|  | **Yes** | **No** |  |
| 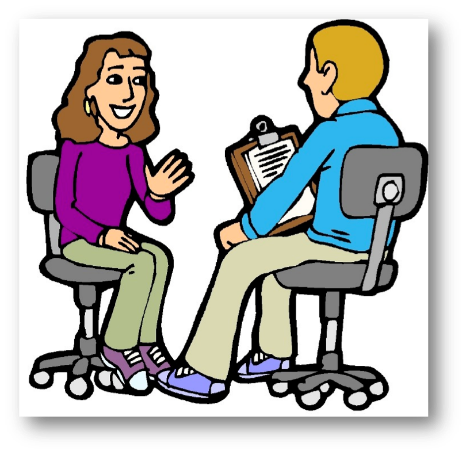 |  |  |  |

| **Picture 2**  **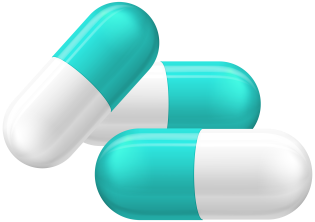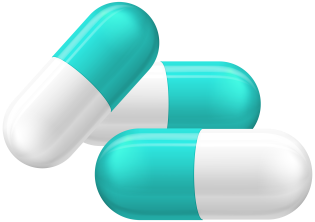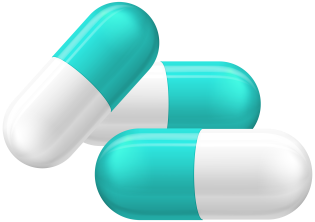** | **Do you understand the picture?** | | **Comment** |
| --- | --- | --- | --- |
|  | **Yes** | **No** |  |
| 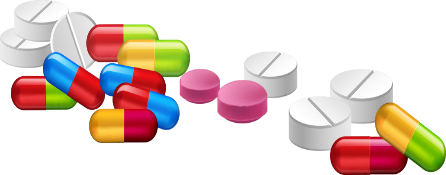 |  |  |  |

| **Picture 3**  **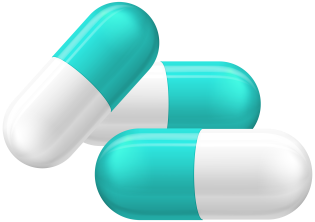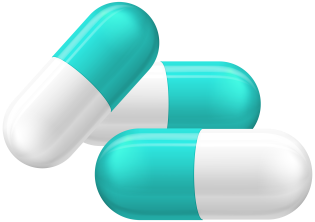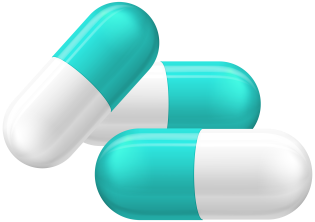** | **Do you understand the picture?** | | **Comment** |
| --- | --- | --- | --- |
|  | **Yes** | **No** |  |
| **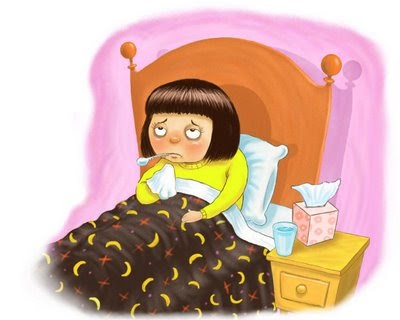** |  |  |  |

| **Picture 4**  **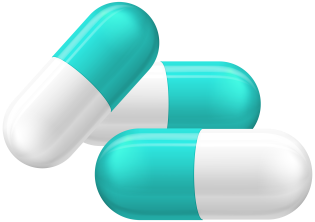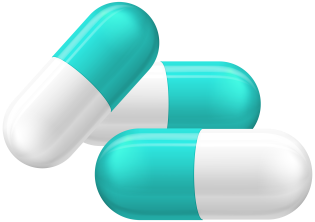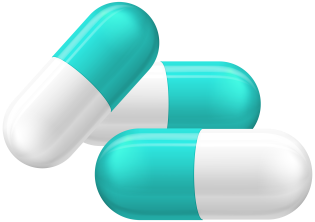** | **Do you understand the picture?** | | **Comment** |
| --- | --- | --- | --- |
|  | **Yes** | **No** |  |
| **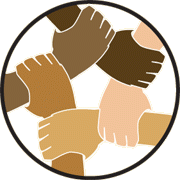** |  |  |  |

| **Picture 5**  **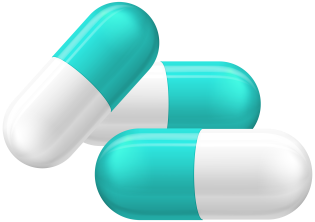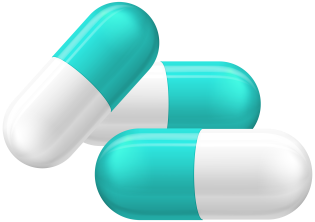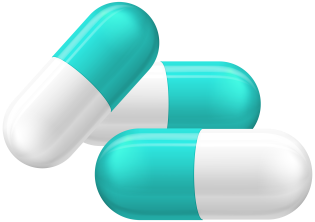** | **Do you understand the picture?** | | **Comment** |
| --- | --- | --- | --- |
|  | **Yes** | **No** |  |
| **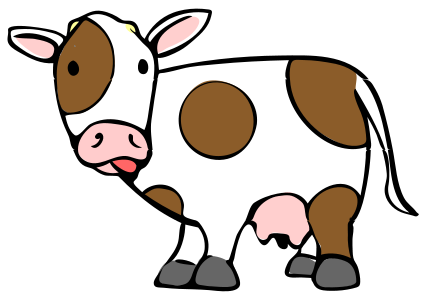**  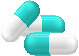 |  |  |  |

| **Picture 6**  **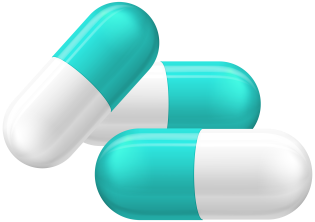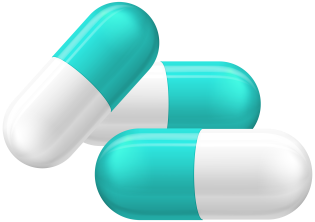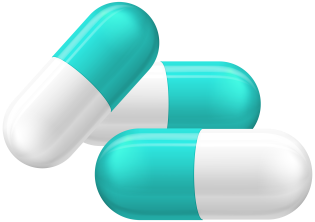** | **Do you understand the picture?** | | **Comment** |
| --- | --- | --- | --- |
|  | **Yes** | **No** |  |
| **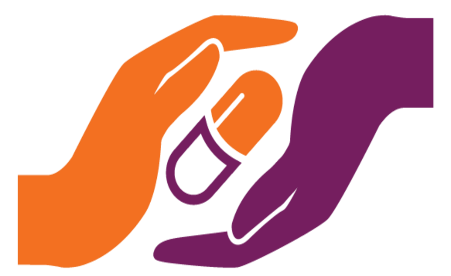** |  |  |  |

| **Picture 7**  **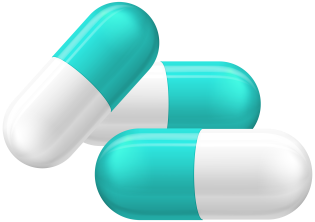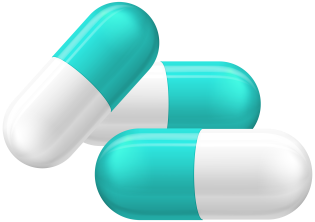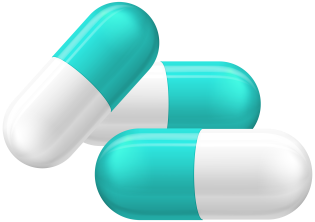** | **Do you understand the picture?** | | **Comment** |
| --- | --- | --- | --- |
|  | **Yes** | **No** |  |
| **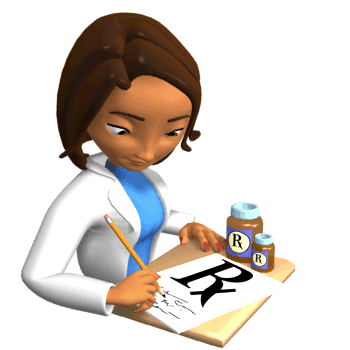** |  |  |  |

| **Picture 8**  **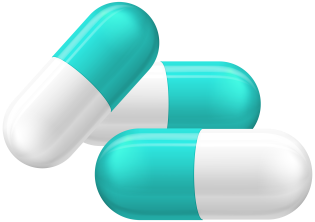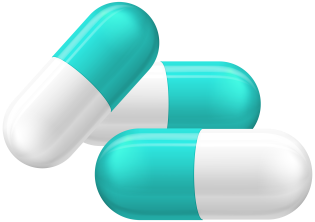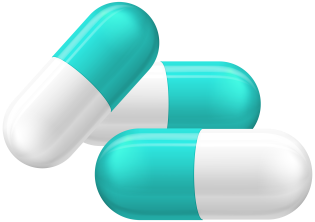** | **Do you understand the picture?** | | **Comment** |
| --- | --- | --- | --- |
|  | **Yes** | **No** |  |
| **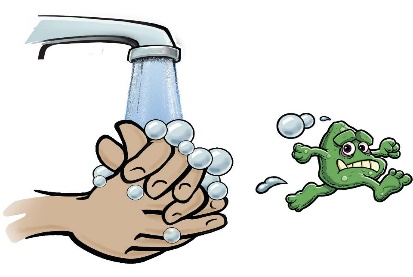** |  |  |  |

| **Picture 9**  **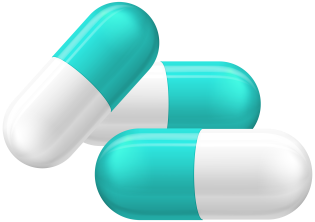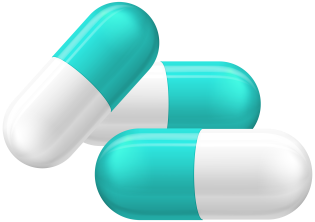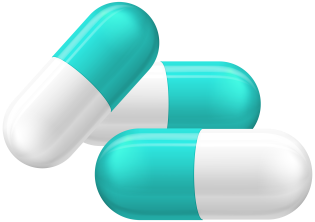** | **Do you understand the picture?** | | **Comment** |
| --- | --- | --- | --- |
|  | **Yes** | **No** |  |
| **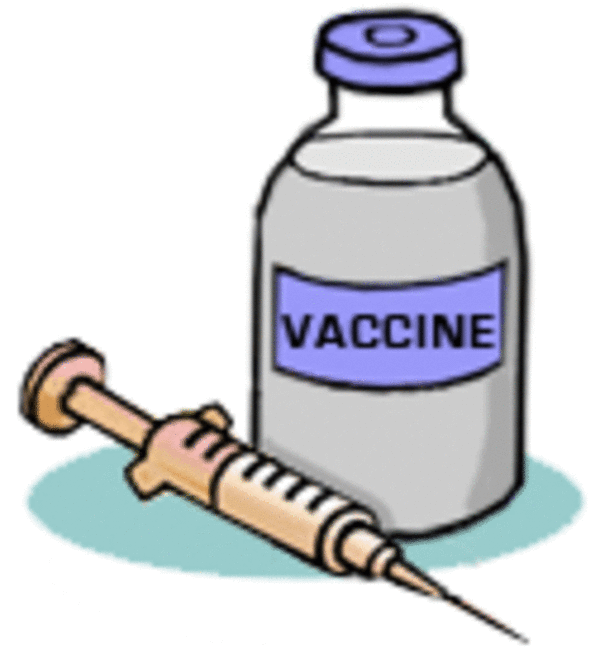** |  |  |  |


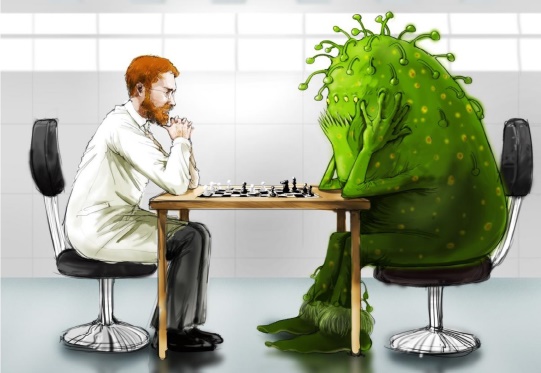

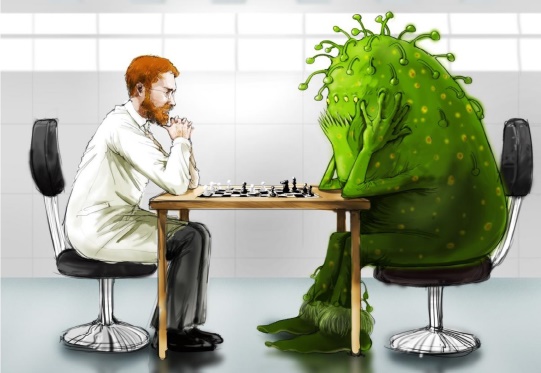


1. Did you understand all the pictures?

| **Yes** | **No** |
| --- | --- |
|  |  |

If your answer to the above question is “No”, please state the number/s of the picture/s which you did not understand and explain why.

………………………………………………………………………………………………………………………………………………………………………………………………………………………………………………………………………………………………………………………………………………………………………………………………………………………………………………………………………………………………………………………………………………………………………………………………………………………

1. Where any of the pictures helpful to you?

| **Yes** | **No** |
| --- | --- |
|  |  |

If your answer to the above question is “Yes”, please state the number/s of the picture/s which you found helpful and explain why.

………………………………………………………………………………………………………………………………………………………………………………………………………………………………………………………………………………………………………………………………………………………………………………………………………………………………………………………………………………………………………………………………………………………………………………………………………………………

1. Are any of the pictures culturally or religiously offensive to you?

| **Yes** | **No** |
| --- | --- |
|  |  |

If your answer to the above question is “Yes”, please state the number/s of the picture/s which you found offensive and explain why.

………………………………………………………………………………………………………………………………………………………………………………………………………………………………………………………………………………………………………………………………………………………………………………………………………………………………………………………………………………………………………………………………………………………………………………………………………………………

1. Would you think about the number of pictures in the leaflet?

| **Too much** | **Just right** | **Too little** |
| --- | --- | --- |
|  |  |  |

**Leaflet Assessment**

1. Is the information in the leaflet easy to understand?

| **Yes** | **No** |
| --- | --- |
|  |  |

Please explain your answer to the above question.

………………………………………………………………………………………………………………………………………………………………………………………………………………………………………………………………………………………………………………………………………………………………………

1. Is the font easy to read?

| **Yes** | **No** |
| --- | --- |
|  |  |

Please explain your answer to the above question.

………………………………………………………………………………………………………………………………………………………………………………………………………………………………………………………………………………………………………………………………………………………………………

1. What do you think about the amount of text in the leaflet?

| **Too much** | **Just right** | **Too little** |
| --- | --- | --- |
|  |  |  |

Please explain if your answer to the above question is “Too much” or “Too little”.

………………………………………………………………………………………………………………………………………………………………………………………………………………………………………………………………………………………………………………………………………………………………………

1. Were you able to understand all of the words in the leaflet?

| **Yes** | **No** |
| --- | --- |
|  |  |

If your answer to the above question is “No”, please list the words which you did not understand, and/or circle the words on the leaflet itself.

………………………………………………………………………………………………………………………………………………………………………………………………………………………………………………………………………………………………………………………………………………………………………

1. Is the language easy to understand?

| **Yes** | **No** |
| --- | --- |
|  |  |

Please explain your answer to the above question.

………………………………………………………………………………………………………………………………………………………………………………………………………………………………………………………………………………………………………………………………………………………………………

1. Is there any further information that you think is required?

| **Yes** | **No** |
| --- | --- |
|  |  |

Please explain your answer to the above question.

………………………………………………………………………………………………………………………………………………………………………………………………………………………………………………………………………………………………………………………………………………………………………………………………………………………………………………………………………………………………………………………………………………………………………………………………………………………

1. To what extent does this leaflet help you understand the causes, consequences and prevention methods of Antimicrobial Resistance?

| **Very helpful** | **Relatively helpful** | **Not helpful** |
| --- | --- | --- |
|  |  |  |

1. Do you have any further comments and/or suggestions?

………………………………………………………………………………………………………………………………………………………………………………………………………………………………………………………………………………………………………………………………………………………………………………………………………………………………………………………………………………………………………………………………………………………………………………

**Thank you for your participation in this assessment!**

## **Pre- and Post-Workshop Questionnaire (Workshop 1)**

Participant Number..........................................

Age..........................................

Gender..........................................

Home Language..........................................

Date..........................................

**Post-Workshop Questionnaire: Antimicrobial Resistance**

Please indicate your answer by ticking under “Yes” or “No”, or circling the correct answer (a, b, c or d). Please note that there is only ONE correct answer for each question.

1. Are all types of microbes (bacteria, viruses, fungi, parasites) bad for health?

| **Yes** | **No** |
| --- | --- |
|  |  |

1. Antibiotics are medicines that help to fight __________

a) viruses

b) bacteria

c) fungi

d) parasites

1. Can antibiotics help to fight the flu?

| **Yes** | **No** |
| --- | --- |
|  |  |

1. Antibiotic resistance is when antibiotics can _______________

a) fight and kill bacteria

b) **no longer** fight and kill bacteria

c) fight and kill all microbes

d) **no longer** fight and kill all microbes

1. What can cause antibiotic resistance?

a) Using the antibiotics exactly as given by the doctor or nurse at the clinic

b) Keeping the place clean

c) Finishing the antibiotic course

d) Sharing and using leftover antibiotics

1. Can resistant bacteria be treated with the same antibiotic?

| **Yes** | **No** |
| --- | --- |
|  |  |

1. Can we get microbes from the things we touch?

| **Yes** | **No** |
| --- | --- |
|  |  |

1. Can we remove all microbes by washing our hands with water alone?

| **Yes** | **No** |
| --- | --- |
|  |  |

1. We should wash our hands before _______________

a) using the toilet

b) cooking food

c) coughing

d) blowing our nose

1. What will happen if antibiotic resistance keeps increasing?

a) There will be more deaths

b) We will not get sick

c) We will spend less nights staying in the hospital

d) We will spend less money on medicines

1. How can we control and stop antibiotic resistance?

a) Get antibiotics from the clinic as soon as we get the flu or cold

b) Share antibiotics with our families and friends when they are sick

c) Using the antibiotics exactly as given by the doctor or nurse

d) Stop taking antibiotics once we feel better

1. Do you have any further comments and/or suggestions?

………………………………………………………………………………………………………………………………………………………………………………………………………………………………………………………………………………………………………………………………………………………………………………………………………………………………………………………………………………………………………………………………………………………………………………

**Thank you for your participation in this assessment!**

## **Pre- and Post-Workshop Questionnaire (Workshop 2)**

Participant Number..........................................

Age..........................................

Gender..........................................

Home Language..........................................

Education Level..........................................

Job Title..........................................

Institution/Clinic..........................................

Date..........................................

**Pre- and Post-Workshop Questionnaire on Antimicrobial Resistance**

Please indicate your answer by ticking under “Yes” or “No”, or circling the correct answer (a, b, c, or d). Please note that there is only ONE correct answer for each question.

1. Antimicrobial resistance is when the correct medicine can no longer fight the infection.

| **Yes** | **No** |
| --- | --- |
|  |  |

1. Antimicrobial resistance **only** occurs in patients over 65 years.

| **Yes** | **No** |
| --- | --- |
|  |  |

1. What will happen if antibiotic resistance keeps increasing?

a) There will be more deaths

b) We will not get sick

c) We will spend less nights staying in the hospital

d) We will spend less money on medicines

1. What can cause antibiotic resistance?

|  | **Yes** | **No** |
| --- | --- | --- |
| Using the antibiotics exactly as prescribed by the doctor or nurse at the clinic |  |  |
| Finishing the antibiotic course |  |  |
| Sharing antibiotics with others |  |  |
| Using leftover antibiotics |  |  |
| Keeping the place clean |  |  |

1. Can we remove all harmful microbes by washing our hands with water alone?

| **Yes** | **No** |
| --- | --- |
|  |  |

1. We should wash our hands **before** _______________

a) using the toilet

b) cooking food

c) coughing

d) blowing our nose

1. How can we control and manage the increasing antibiotic resistance?

|  | **Yes** | **No** |
| --- | --- | --- |
| Get antibiotics from the clinic as soon as we get the flu or cold |  |  |
| Stop taking antibiotics once we feel better |  |  |
| Share antibiotics with our families and friends when they are sick |  |  |
| Using the antibiotics exactly as prescribed by the doctor or nurse at the clinic |  |  |

1. Do you have any further comments and/or suggestions?

………………………………………………………………………………………………………………………………………………………………………………………………………………………………………………………………………………………………………………………………………………………………………………………………………………………………………………………………………………………………………………………………………………………………………………

**Thank you for your participation in this assessment!**

## **Feedback Sheet for AMR Trainer’s Manual**

Date..........................................

**Feedback Sheet: Workshop for Trainer’s Manual on Antimicrobial Resistance**

Please form groups with five people in each group. Please can each person in the group fill in the table below:

| **Participant Number** | **Age** | **Gender** | **Home Language** | **Education Level** | **Job Title** | **Institution or Clinic** |
| --- | --- | --- | --- | --- | --- | --- |
|  |  |  |  |  |  |  |
|  |  |  |  |  |  |  |
|  |  |  |  |  |  |  |
|  |  |  |  |  |  |  |
|  |  |  |  |  |  |  |

**Please choose a team leader in each group.** After the group discussion, the team leader has to report back on the following aspects of the trainer’s manual:

- Content comprehension: What do you understand from this section of the trainer’s manual? What is the key message/s from this section of the trainer’s manual? Do you understand the words and pictures used in the trainer’s manual?
- Culture-sensitivity: Do you find any of the information or pictures in this section of the trainer’s manual offensive? If your answer is yes, please discuss why.
- Personal opinions: What information and/or pictures need to be changed? What additional information is needed?
- Discuss 3 – 5 points on how your daily standard practices have changed after using the trainer’s manual and based on these workshops.

**Thank you for your participation in this feedback session!**

## **Final AMR Trainer’s Manual (English and IsiXhosa)**

| 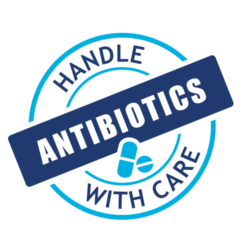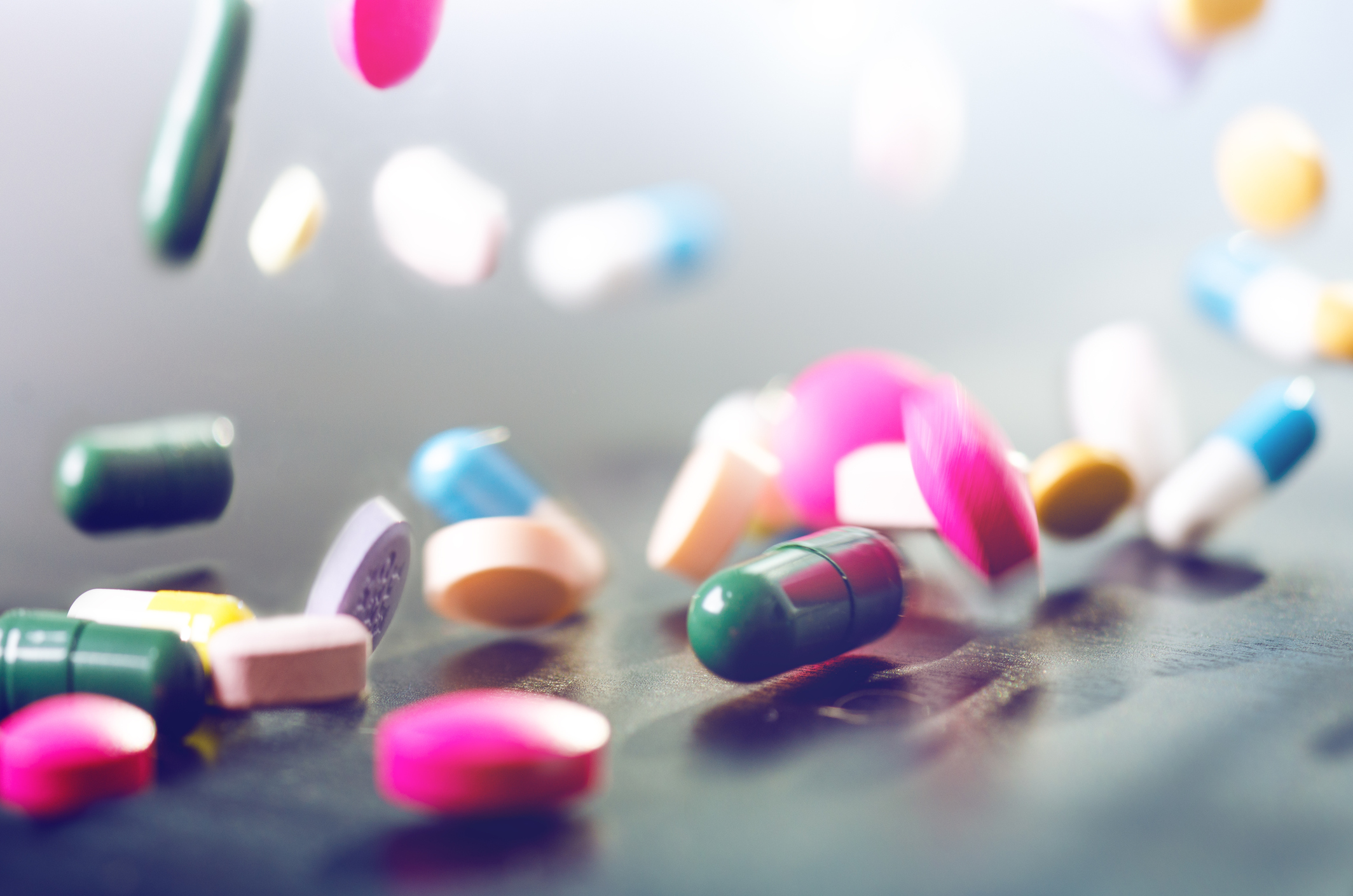 |
| --- |
|  |
| \| Samridhi Sharma, Sunitha Srinivas and Roman Tandlich \| Faculty of Pharmacy,  Rhodes University \|  \| \| --- \| --- \| --- \| |

**Table of Contents**

[**Preface** 246](#_Toc125734770)

[**Intshayelelo** 246](#_Toc125734771)

[**Microbes** 247](#_Toc125734772)

[Learning Outcomes 247](#_Toc125734773)

[**Intsolongwane** 247](#_Toc125734774)

[IziPhumo zokuFunda 247](#_Toc125734775)

[What are Microbes? 248](#_Toc125734776)

[Zintoni Iintsholongwane? 248](#_Toc125734777)

[Good Microbes vs Bad Microbes 249](#_Toc125734778)

[Intsholongwane Ezilungileyo vs Iintsolongwane Ezingalunganga 249](#_Toc125734779)

[Types of Microbes (Iintlobo zentsholongwane) 250](#_Toc125734780)

[**Antimicrobial Resistance** 253](#_Toc125734781)

[Learning Outcomes 253](#_Toc125734782)

[IziPhumo zokuFunda 253](#_Toc125734783)

[What are Antibiotics? 253](#_Toc125734784)

[Yintoni isibulala-ntsholongwane? 253](#_Toc125734785)

[What is Antimicrobial Resistance? 255](#_Toc125734786)

[Impact of Antimicrobial Resistance 256](#_Toc125734787)

[Impembelelo yokuchasa izibulali-ntsholongwane 256](#_Toc125734788)

[Causes of Antimicrobial Resistance (Unobangela wochaso kwamayeza entsholongwane) 258](#_Toc125734789)

[**Hand Hygiene** 258](#_Toc125734790)

[Learning Outcomes 258](#_Toc125734791)

[Why Should You Wash Your Hands? 258](#_Toc125734792)

[**Ukucoceka kwezandla** 258](#_Toc125734793)

[IziPhumo zokuFunda 258](#_Toc125734794)

[Kutheni Ufanele Uhlambe Izandla zakho? 259](#_Toc125734795)

[When Should You Wash Your Hands? (Kufuneka uzivase nini izandla zakho?) 259](#_Toc125734796)

[How Should You Wash Your Hands? (Kufuneka uzihlambe njani izandla zakho?) 260](#_Toc125734797)

[**How Can You Prevent and Control the Spread of AMR?** 261](#_Toc125734798)

[**Ungathintela njani kwaye ulawule ukwanda kwe-AMR?** 261](#_Toc125734799)

[**Workshop Activity on the Importance of Hand Hygiene (Umsebenzi ngokubaluleka kokucocwa kwezandla)** 262](#_Toc125734800)

**Preface**

A mother comes into a health-care facility when her child has a high fever, hoping that the child will get effective treatment and be cured. With increasing rates of antimicrobial resistance (AMR), treatment options reduce, and her hopes may be crushed if the bacteria have become resistant and available antibiotics no longer work.

This trainer’s manual is meant to help increase awareness on AMR. It provides practical guidance to support the implementation of the first objective of the World Health Organization (WHO) Global Action Plan on AMR: to improve awareness and understanding of AMR.

**Intshayelelo**

Umama ungena kwindawo yokhathalelo lwempilo xa umntwana wakhe enomkhuhlane omkhulu, ngethemba lokuba umntwana uya kufumana unyango olufanelekileyo kwaye anyangeke. Ngamaqondo akhulayo e-Antimicrobial Resistance (AMR), ukhetho lonyango luyancipha, kwaye amathemba akhe anokuqhekeka ukuba intsholongwana iye ayanyangeka kwaye ukuba kufumanek ukuba amayeza okubulala intsholongwane amasasebenzi.

Le ncwadana yomqeqeshi yenzelwe ukunceda ukwandisa ulwazi nge-AMR. Inika isikhokelo esisebenzayo sokuxhasa ukuphunyezwa kwenjongo yokuqala ye-World Health Organisation (WHO) IsiCwangciso seNtsebenzo yeHlabathi kwi-AMR: ukuphucula ulwazi kunye nokuqonda kwe-AMR.

**Microbes**

**Learning Outcomes**

- There are five different types of microbes – bacteria, viruses, fungi, helminths and protozoa
- Some microbes are useful, but some can be harmful and cause infections
- Antimicrobials are medicines used to treat infections caused by microbes

**Intsolongwane**

**IziPhumo zokuFunda**

- Kukho iintlobo ezintlanu ezahlukeneyo zentsholongwane - ibacteria, ivirus, ifungi, i-helminths kunye ne-protozoa
- Ezinye iintsholongwane ziluncedo, kodwa ezinye zinokuba yingozi kwaye zibangele usulelo
- I-antimicrobials ngamayeza asetyenziswa ukunyanga usulelo olubangelwa ziintsholongwane

**What are Microbes?**

**Zintoni Iintsholongwane?**

**Good Microbes vs Bad Microbes**

**Intsholongwane Ezilungileyo vs Iintsolongwane Ezingalunganga**

| Good Microbes  (Intsholongwane ezilungileyo) | Bad Microbes  (Iintsolongwane ezingalunganga) |
| --- | --- |
| Do **not** cause disease  Azibangeli zifo | Some microbes can be **harmful** to humans and cause disease or illness  Ezinye iintsholongwane zinokuba **yingozi** ebantwini kwaye zibangele izifo okanye ukugula |
| Responsible for making foods such as wine, cheese, vinegar, yogurt, and chocolate  Zinoxanduva lokwenza ukutya okufana newayini, isonka samasi, iviniga, iyoghurt kunye netshokholethi | Known as **pathogens** but are sometimes called ‘bugs’ or ‘germs’  Zaziwa ngokuba ziintsholongwane kodwa ngamanye amaxesha zibizwa ngokuba zi 'bugs' okanye 'germs' |
| Used for probiotics to help keep the gut healthy  Zisetyenziselwa ii-probiotic ukunceda ukugcina isisu sisempilweni | Pathogens can spread by close contact, coughs, sneezes, food, water and animals  Iintsholongwane zisasazeka ngokusondellana kwabantu, ukukhohlela, ukuthimla, ukutya, amanzi kunye nezilwanyana |
| Help in digestion  Zinceda ekugayeni | Grow in unhygienic or dirty environments – on humans, their home, schools etc.  Zikhula kwindawo engathandekiyo okanye emdaka-ebantwini, ekhaya, kwizikolo njl |

**Types of Microbes (Iintlobo zentsholongwane)**

There are five different types of microbes (Zintlanu iintlobo zentsholongwane):


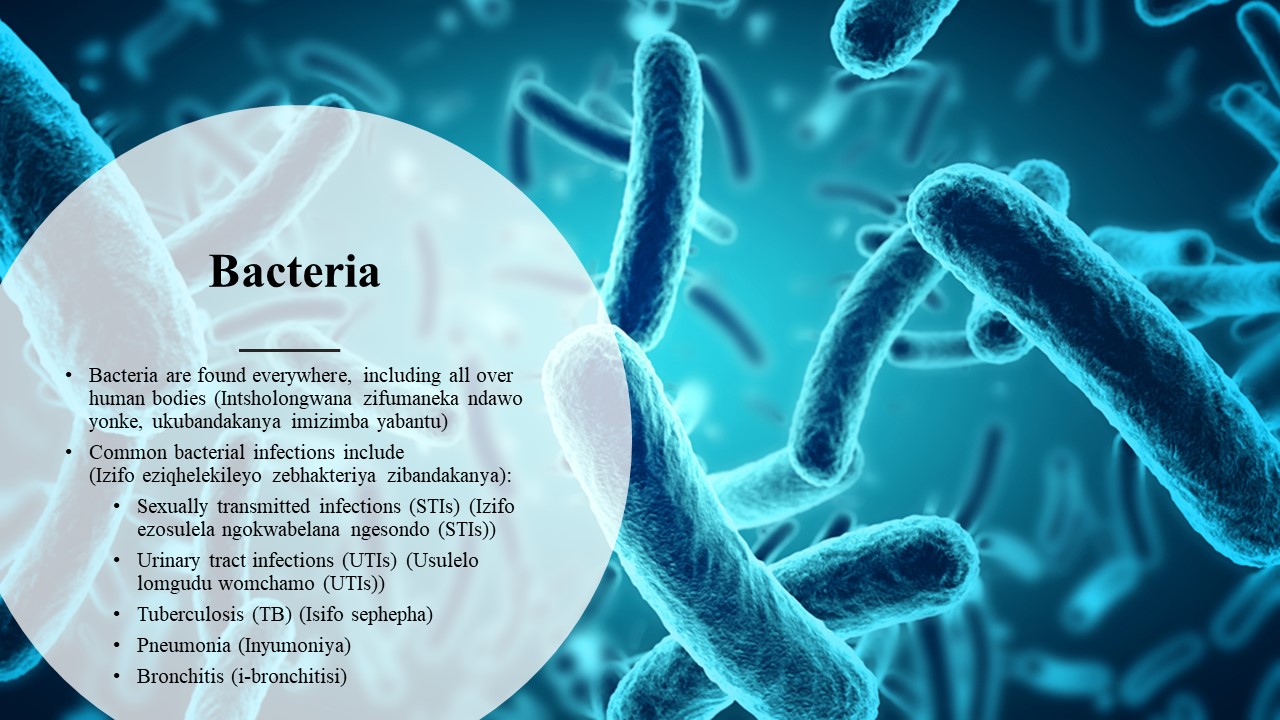


*Figure 1: Bacterial cells; no copyright required*


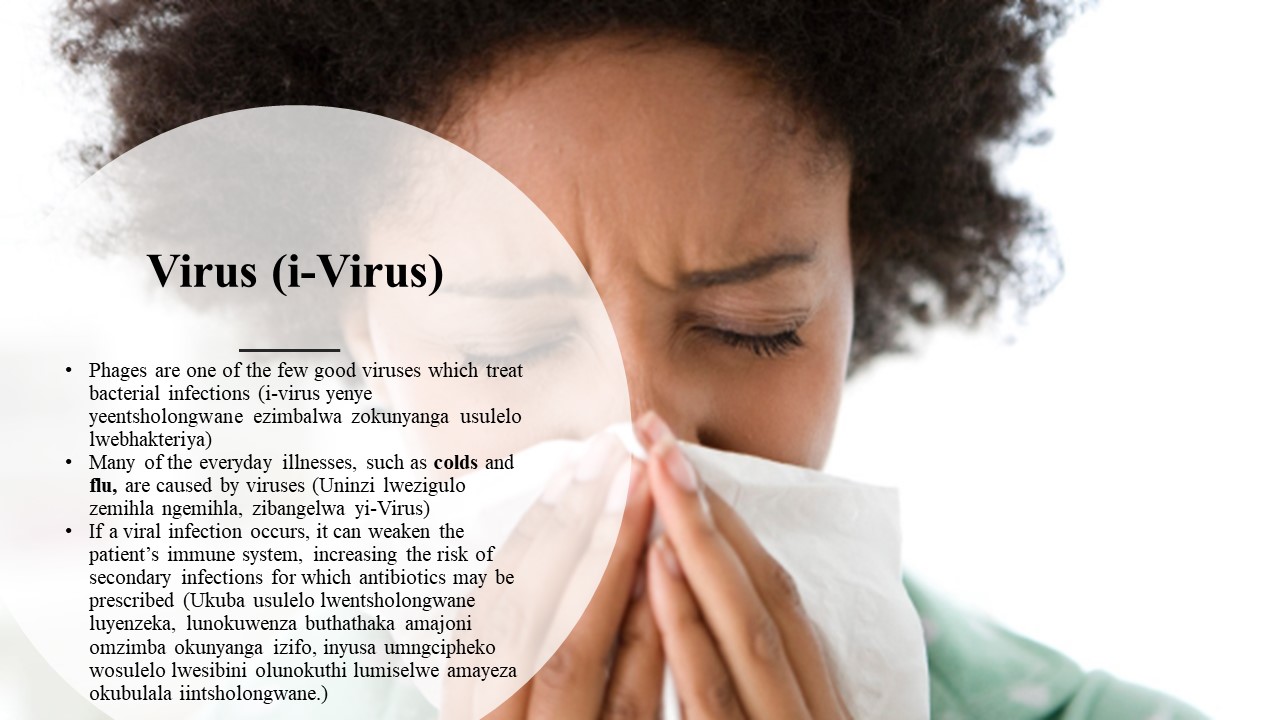


*Figure 2: A person with a viral infection i.e. a cold/flu; no copyright required*


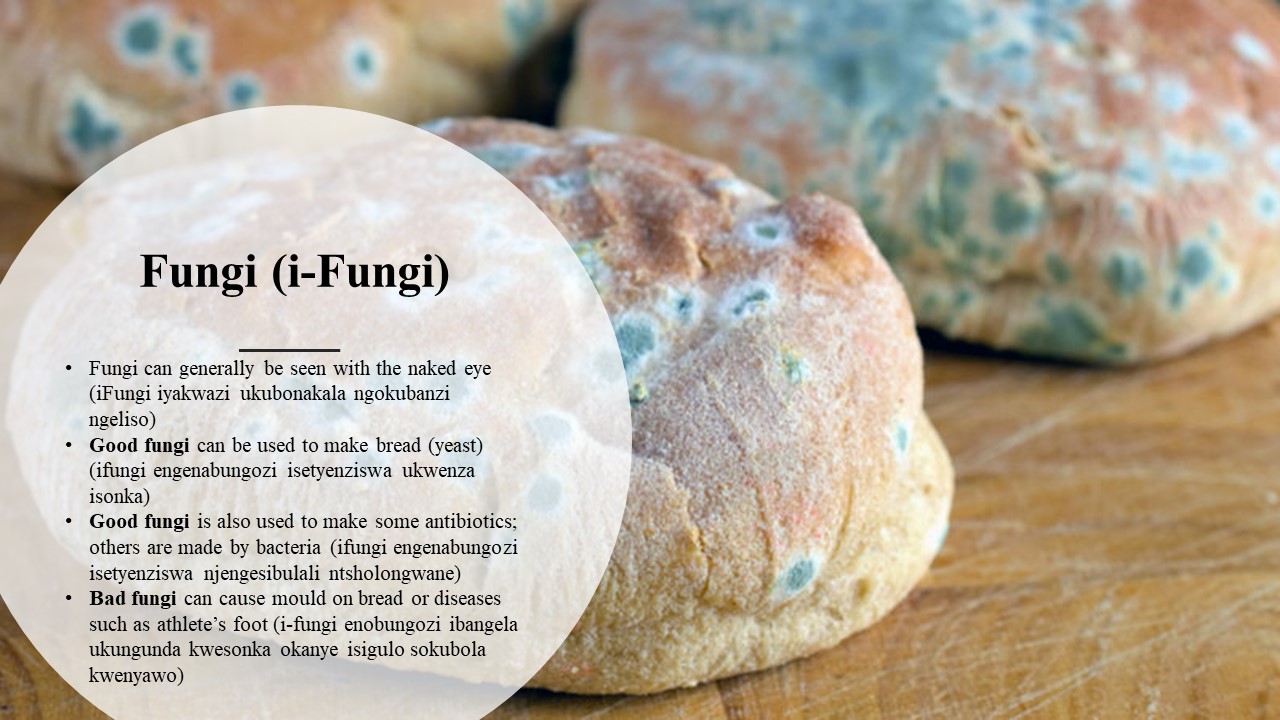


*Figure 3: Mould (bad fungi) on bread; no copyright required*


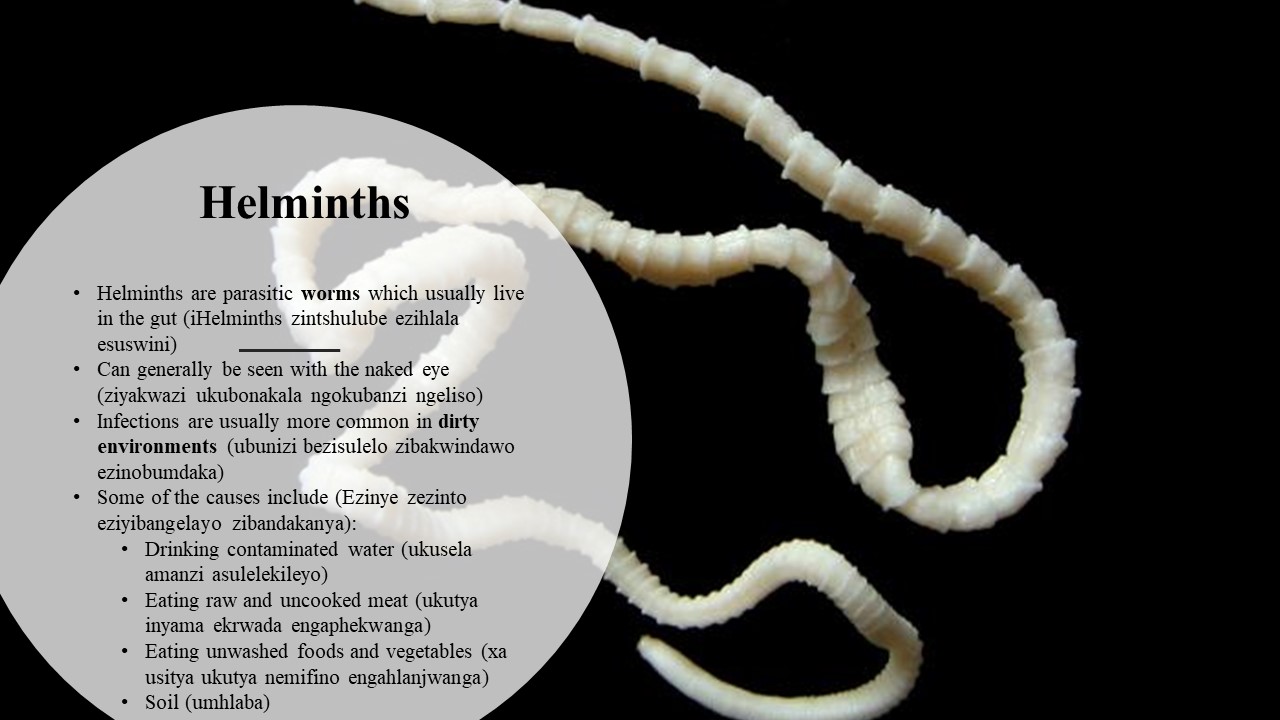


*Figure 4: Helminths i.e. worms; no copyright required*


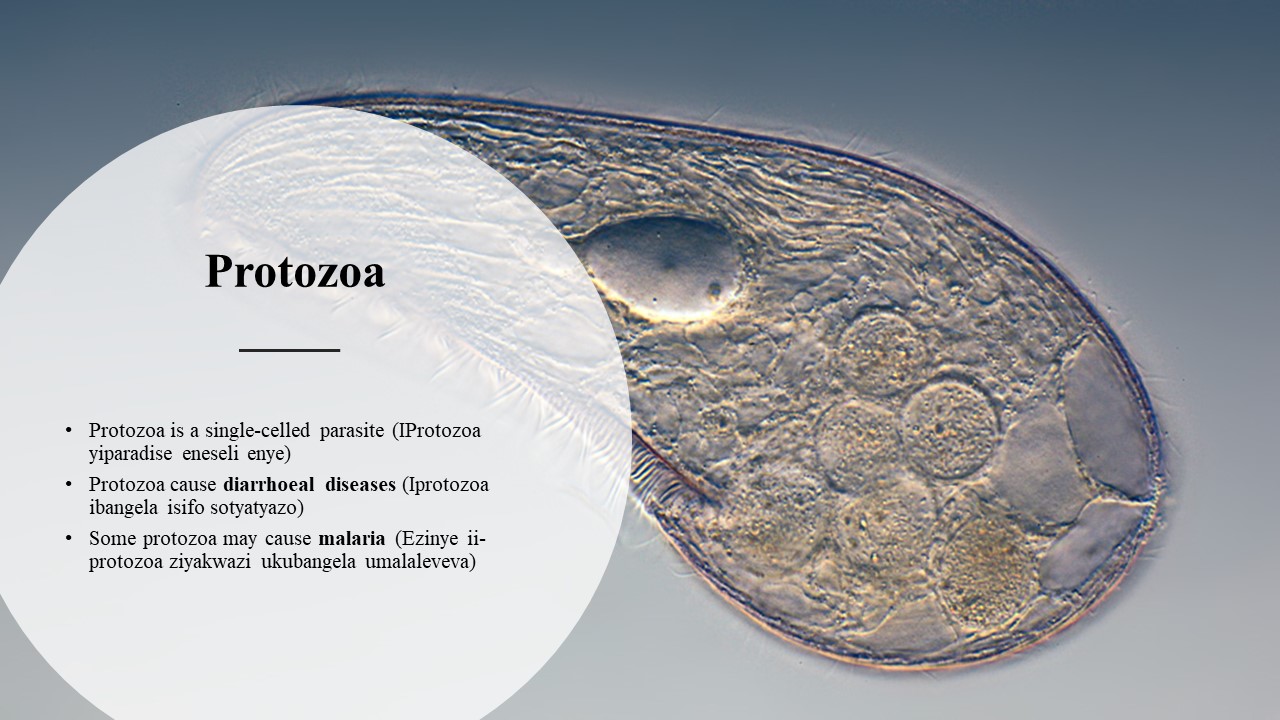


*Figure 5: A close-up of a protozoal cell; no copyright required*

**Antimicrobial Resistance**

**Learning Outcomes**

- Antibiotics are medicines that only work on bacteria
- Antibiotics do not work on viral infections like most coughs, colds and flu
- Bacteria are becoming resistant to antibiotics
- Individuals can help prevent more bacteria from becoming resistant to antibiotics

**IziPhumo zokuFunda**

- Ii-antibiotics ngamayeza okubulala iintsholongwane
- Ii-antibiotics azisebenzi kulosuleleko lwe-virus njengokukhohlela okuninzi, isigulo sengqele kunye nomkhuhlane
- Iibacteria ziya zinganyangeki kwaye ziyaxhathisa ekubeni zibulaleke ngalamayeza
- Abantu aba bodwa banokunceda ukuthintela ii-bacteria ezininzi ekubeni zinganyangeki ngalamayeza

**What are Antibiotics?**

-
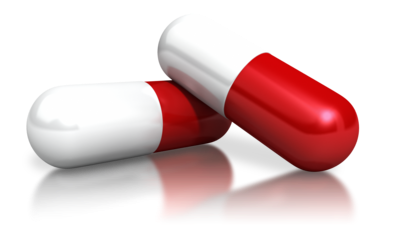
Most of the time our immune system can fight against harmful microbes but sometimes it needs help
- Some microbes can cause serious and sometimes fatal infections
- Bacteria are a type of microbe that can cause some of these infections i.e. bacterial infections
- **Antibiotics** are medicines which kill bacteria and make us feel better if we have a bacterial infection
- When antibiotics are taken with **caffeine**, some of their common side effects, such as restlessness, headache, nausea and vomiting, can become worse.

**Yintoni isibulala-ntsholongwane?**

- Ixesha elininzi amajoni ethu omzimba ayakwazi ukulwa neentsholongwane ezinobungozi kodwa ngamanye amaxesha ifuna uncedo
- Ezinye iintsholongwane zinokubangela usulelo olunobungozi kwaye ngamanye amaxesha usulelo olubulalayo
- Ibacteria luhlobo lwentsholongwane olunokubangela ezinye zezi zifo umzekelo: ulosuleleko lwebhakteriya
- Izibulala-ntsholongwane ngamayeza abulala iibhaktheriya kwaye asenza sizive ngcono ukuba sinosulelo lwebhaktiriya
- Xa izibulala ntsholongwane zithathwa nezidakamiswa ezifana ne-caffeine, ezinye zeziphumo ezifana nokuqaqanjelwa yintloko, isicaphucaphu nokugabha, kunokuphangalala.

*An* ***Antibiotic*** *is an antimicrobial agent that kills or inhibits the growth of bacteria (isibulala-ntsholongwane sibulala ukukhula kwebhaktheriya)*

An antimicrobial is an agent that fights against any type of microbe, such as bacteria, viruses, fungi and parasites. As shown in the diagram below, all antibiotics are antimicrobials, but not all antimicrobials are antibiotics.

Isibulali-ntsholongwane yiarhente elwa ngokuchasana noluphi na uhlobo lwe-ntsholongwane, enjengebhakteriya, ivirus, ifungi kunye neparasites. Njengoko kubonisiwe kumzobo ongezantsi, zonke iiantibiotics zi-antimicrobials, kodwa ayisizizo zonke ii-antimicrobials eziyi antibiotic.

**What is Antimicrobial Resistance?**

**
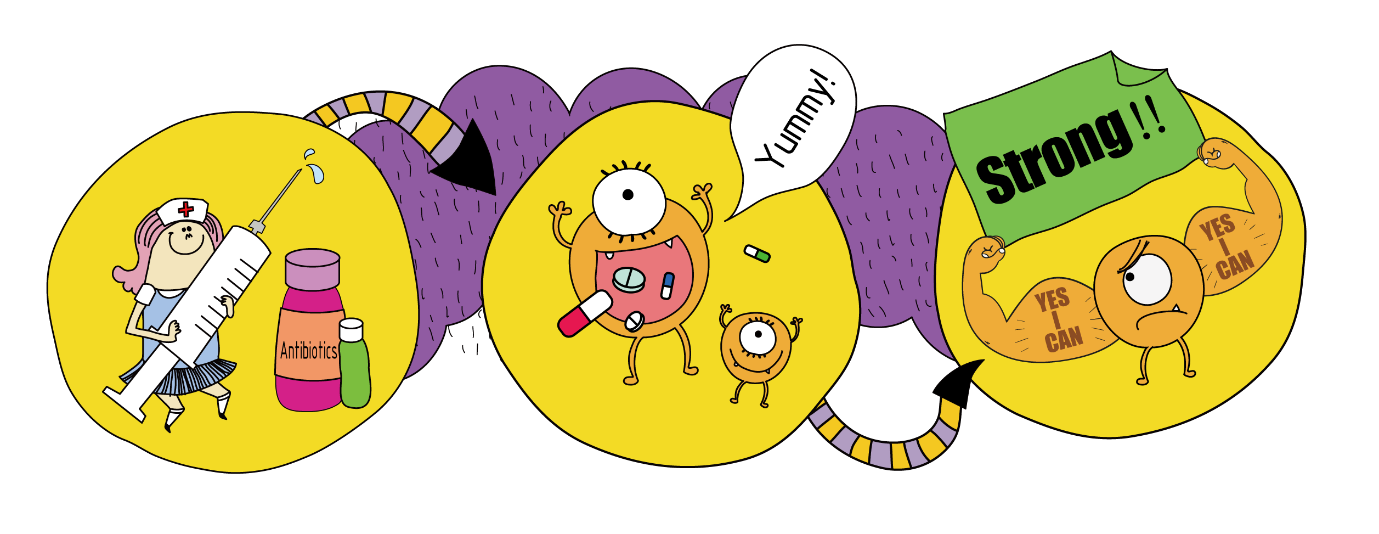
**

***Antimicrobial Resistance*** *is when microbes (e.g. bacteria and viruses) no longer respond to the appropriate medicines (e.g. antibiotics and antivirals) used to treat infections. Antimicrobial Resistance can affect any race, sex, or age. (Ukuchasana ne-antimicrobial kuxa iintsholongwane (umz. Ibhakteriya nevirus) zingasakwazi ukubulaleka nangeliphina iyeza (umz. Antibiotics kunye ne-antiviral) elisetyenziswa ukubulala usulelo. Ukuchasana ne-antimicrobial kunokuchaphazela naluphi na uhlanga, isini, okanye ubudala.)*

- The microbes become resistant to the medicine
  - E.g. bacteria, not humans or animals, become resistant to the antibiotic
- A growing list of infections – such as pneumonia, tuberculosis, sexually transmitted infections (STIs), and foodborne diseases – are becoming harder, and sometimes impossible, to treat because antibiotics are becoming less effective
- If urgent action is not taken to tackle AMR, common infections and small injuries can once again cause death.^^[[1]](#footnote-1)^^
- Untreated infections can spread to others
- Without effective antimicrobials for preventing and treating infections, medical procedures such as organ transplants, cancer chemotherapy, diabetes management and major surgery (e.g. c-sections) become very high risk
- Iintsholongwane ziyakwazi ukuchasana namayeza
- Umz. ibhakteriya, hayi abantu okanye izilwanyana, ziyachasana neyeza
- Uluhlu olukhulayo losulelo – njengenyumoniya, isifo sephepha, izifo ezosulela ngokwabelana ngesondo (STIs), kunye nezifo ezibangelwa kukutya- ziya ziba nzima, kwaye ngamanye amaxesha akunakwenzeka, ukunyanga kuba amayeza okubulala iintsholongwane aya engasebenzi ukuzibulala
- Ukuba inyathelo elingxamisekileyo alithathwanga ukujonganew ne-AMR, usulelo oluqhelekileyo kunye nokulimala okuncinci kunokubangela ukufa.^1^
- Usulelo olunganyangekiyo lunokusasazeka kwabanye
- Ngaphandle kokusebenza okukuko okukuko kokuthintela nokunyanga usulelo, iinkqubo zonyango ezinjengokudluliselwa kwamalungu omziba, unyango lomhlaza, ukuphathwa sisifo seswekile kunye nonyango olukhulu (umz. Uqhaqho lwesibeleko) lubasemngciphekweni omkhulu

**Impact of Antimicrobial Resistance**

- Antimicrobial Resistance currently causes 700,000 global deaths per year
- By 2050, Antimicrobial Resistance will cause up to 10 million global deaths per year, out of which 4,150,000 deaths will be in Africa
- Drug resistance is starting to complicate the fight against TB and HIV
- Resistance in TB:
  - Multidrug-resistant tuberculosis (MDR-TB) is a form of tuberculosis that is resistant to the two most powerful anti-TB medicines. MDR-TB requires treatment courses that are much longer and less effective than those for non-resistant TB.
  - Extensively drug-resistant tuberculosis (XDR-TB) is a form of tuberculosis that is resistant to at least four of the core anti-TB medicines. About 10% of people with MDR-TB have XDR-TB.
- Resistance in HIV: The second-line treatment is 3 times more and third-line treatment is 18 times more expensive than first-line treatments.^^[[2]](#footnote-2)^^ Having too much caffeine can also change how HIV treatments work in the human body and can have side effects. Caffeine consumption results in increased periods of not being able to sleep i.e. insomnia, which could worsen HIV.^^[[3]](#footnote-3)^^
- If AMR is not tackled:

**Impembelelo yokuchasa izibulali-ntsholongwane**

- Ukuchasana nezibulali ntsholongwane okwangoku kubangela ukuba abantu abangama-700,000 basweleke kwihlabathi ngonyaka
- Ngo-2050, ukuchasa izibulali-ntsholongwane kuya kubangela ukufa kwezigidi ezili-10 ngonyaka, kuthi apho abantu abayi-4,150,000 bayokufa e-Afrika.
- Ukuxhathisa kweentsholongwane kwiziyobisi ezenzelwe ukuzibulala kuya kuqala ukwenza nzima umlo ngokuchasene neTB kunye ne-HIV
- Ukuchasa kwe-TB:
- Isifo sephepha esinganyangekiyo ngamachiza namayeza (i-MDR-TB) luhlobo lwesifo sephepha esichasene namayeza amabini anamandla okulwa i-TB. I-MDR-TB ifuna iikhosi zonyango ezide kwaye zingasebenzi kakuhle kunezo zeTB ezinyangekayo.
- Isifo esinganyangekiyo kakhulu ngamachiza esifo sephepha (i-XDR-TB) luhlobo lwesifo sephepha esinganyangeki okungenani kumayeza amane aphambili anyanga iTB. Malunga ne-10% yabantu abane-MDR-TB bane-XDR-TB.
- Ukuchasa kwe-HIV: Unyango lodidi lwesibini luphindaphindwe amaxesha ama-3 kwaye unyango lomgca wesithathu luxabisa kakhulu amaxesha ayi-18 kunonyango lodidi lokuqala.^2^ Ukuba ne-caffeine eninzi emzimbeni kunakho ukutshintsha ukuba lusebenza njani unyango lwe-HIV emzimbeni womntu kwaye kunokuba nemiphumela emibi. Ukusetyenziswa kweCaffeine kukhokelela ekubeni abantu bangakwazi ukulala i.e. ukuqaqanjelwa, nto leyo enokuyenza ibe mandundu i-HIV.^3^
- Ukuba i-AMR ayijongwanga:

**Causes of Antimicrobial Resistance (Unobangela wochaso kwamayeza entsholongwane)**

Some of the major causes of AMR include (Abanye bonobangela abaphambili be-AMR babandakanya):

**Hand Hygiene**

**Learning Outcomes**

- We pick up microbes from the things we touch and can spread these to other people
- Washing hands can help remove microbes
- Washing hands is one of the best ways to prevent the spread of microbes
- Washing hands with soap and water is better than washing hands with water alone

**Why Should You Wash Your Hands?**

- To remove harmful microbes from your hands and places
- To reduce getting infections
- To prevent the spread of infections
- Washing your hands with **soap and water** is better than washing your hands with water alone

**Ukucoceka kwezandla**

**IziPhumo zokuFunda**

- Sichonga iintsholongwane kwizinto esizichukumisayo kwaye sinokuzisasaza kwabanye abantu
- Ukuhlamba izandla kunokukunceda ususe iintsholongwane
- Ukuhlamba izandla yenye yeendlela ezilungileyo zokuthintela ukusasazeka kweentsholongwane
- Ukuhlamba izandla ngesepha namanzi kungcono kunokuhlamba izandla ngamanzi odwa

**Kutheni Ufanele Uhlambe Izandla zakho?**

- Ukususa iintsholongwane eziyingozi ezandleni zakho nakwi indawo ezahlukileyo
- Ukucutha ukosuleleka
- Ukuthintela ukwanda kosulelo
- Ukuhlamba izandla kunye nesepha namanzi kungcono kunokuhlamba izandla ngamanzi odwa

**When Should You Wash Your Hands? (Kufuneka uzivase nini izandla zakho?)**

**How Should You Wash Your Hands? (Kufuneka uzihlambe njani izandla zakho?)**

Below are the steps to how you should wash your hands (Apha ngezantsi kukho amanyathelo okuba kufuneka uzihlambe njani izandla zakho):

**How Can You Prevent and Control the Spread of AMR?**

- Wash your hands regularly with clean water and soap
- Prepare food hygienically
- Avoid close contact with people when they are ill
- Practice safer sex
- Keep up to date with vaccinations
- Only use antibiotics when prescribed by a doctor or nurse
- Never demand antibiotics if your health worker says you do not need them
- Always follow your health worker’s advice when using antibiotics
- Always finish your treatment course, even if you feel better
- Do not miss any doses
- Do not take double doses when you miss a dose
- Do not share antibiotics with others
- Never use leftover antibiotics

Prepare food hygienically, following the WHO Five Keys to Safer Food (keep clean, separate raw and cooked, cook thoroughly, keep food at safe temperatures, use safe water and raw materials) and choose foods that have been produced without the use of antibiotics for growth promotion or disease prevention in healthy animals.

**Ungathintela njani kwaye ulawule ukwanda kwe-AMR?**

- Hlamba izandla zakho rhoqo ngamanzi acocekileyo kunye nesepha
- Lungisa ukutya ngokucocekile
- Kulumkele ukusondelelana nabantu xa begula
- Yabelana ngesondo ngokukhuselekileyo
- Gcina uvavanyo rhoqo nokudonywa
- Sebenzisa kuphela amayeza okubulala iintsholongwane xa umiselwe ngugqirha okanye umongikazi
- Ungaze ufune i-antibiotics ukuba umsebenzi wakho wezempilo uthi akukho mfuneko
- Landela iingcebiso zomsebenzi wakho wezempilo xa usebenzisa amayeza okubulala iintsholongwane
- Gqiba ikhosi yakho yonyango maxa onke, nokuba uziva ngcono
- Ungaphoswa nawuphina umthamo weyeza
- Musa ukuthatha imithamo yeyeza ephindwe kabini xa uwiphosile umthamo
- Musa ukwabelana ngeentsholongwane nabanye abantu
- Ungaze usebenzise amayeza okubulala iintsholongwane ahleli ithuba elide

Lungisa ukutya ngococeko, ulandela imigaqo emihlanu we-WHO wendlela ezikhuselekileyo zokutya (gcina ucocekile, kwahlule okukrwada nokuphekiweyo, kuphekwe ngokucosekileyo, gcina ukutya kumaqondo obushushu akhuselekileyo, sebenzisa amanzi akhuselekileyo kunye nezinto ezikrwada) kwaye ukhethe ukutya okwenziwe ngaphandle kokusebenzisa amayeza okubulala iintsholongwane ukukhuthaza ukukhula kwako okanye uthintelo lwezifo kwizilwanyana ezisempilweni.

**Workshop Activity on the Importance of Hand Hygiene (Umsebenzi ngokubaluleka kokucocwa kwezandla)**

*Note: This activity aims to show why washing with soap and water is better than using water on its own (Qaphela: Lo msebenzi unenjongo yokubonisa ukuba kutheni ukuhlanjwa kwezandla ngesepha namanzi kungcono kunokusebenzisa amanzi wodwa.).*

**Materials**

- Small bowls
- Water
- Dishwashing liquid
- Fine black pepper

**Izixhobo**

- Izitya ezincinci
- Amanzi
- Isepha yokuhlamba izitya
- Ipepile emnyama

**Set up (Umhlungiselelo)**

1. Set up a few bowls of water with black pepper sprinkled on the surface, a few plain bowls of water, and another bowl with dishwashing liquid in it. (Beka izitya ezimbalwa zamanzi nepepile emnyama efefiweyo phezu kwamanzi, izitya ezimbalwa ezingenanto ngaphandle kwamanzi, kunye nesinye isitya esine-sepha yezitya kuso.)

**Instructions**

1. Tell the participants that the surface of the water in the bowls represents their hands, and that the black pepper represents harmful microbes that need to be washed away.
2. Dip the tip of your finger into the plain bowl of water and then into the pepper water. Gently swirl your finger around and explain that using water to wash your hands only moves the microbes around.
3. Dip the tip of your finger into the bowl of dishwashing liquid and then into the black pepper water.
4. The pepper ‘microbes’ will move towards the edges of the bowl as the soap hits the surface of the water.
5. Tell the group that this shows why using soap when you wash your hands is important, because it breaks up the oils on the surface of your hands that microbes stick to and then they can be rinsed away under running water.^^[[4]](#footnote-4)^^

**Imiyalelo**

1. Xelela abathathi-nxaxheba ukuba umphezulu wamanzi kwizitya umele izandla zabo, kwaye ipepile emnyama imele iintsholongwane ezinobungozi ezifuna ukuhlanjwa.
2. Faka incam yomnwe wakho kwisitya samanzi kwaye uze ungene kumanzi wepepile. Zamisa ngomnwe kancinci uchaze ukuba ukusebenzisa amanzi ukuhlamba izandla kuphela kushukumisa iintsholongwane kuphela, akuzibulali.
3. Faka incam yomnwe wakho kwisitya esinesepha yamanzi gqiba usifake emanzini anepepile.
4. Ipepile 'emele intsholongwane' iya kuhamba ngasemaphethelweni esitya njengoko isepha ihlabe umphezulu wamanzi.
5. Xelela iqela ukuba oku kubonisa ukuba kutheni ukusebenzisa isepha xa uhlamba izandla kubalulekile, kuba kuqhekeza ioyile ebusweni bezandla zakho ezinamathele kwiibhaktheriya kwaye emva koko zinokukhutshwa phantsi kwamanzi abalekayo.


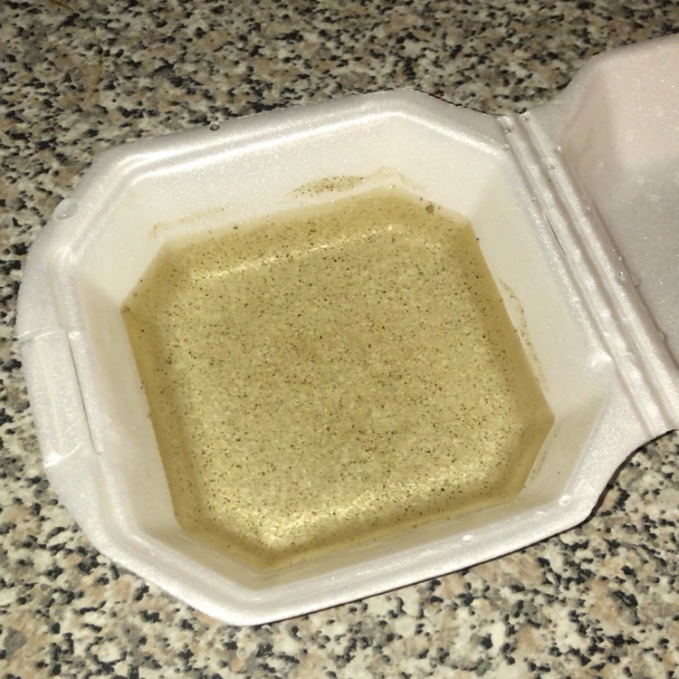


*Figure 6: Display of pepper 'microbes' in water (umboniso wamanzi anepepile (emele intsholongwane))*

1. <https://www.who.int/news-room/fact-sheets/detail/antibiotic-resistance> [↑](#footnote-ref-1)
2. <https://www.who.int/news-room/fact-sheets/detail/antimicrobial-resistance> [↑](#footnote-ref-2)
3. <https://www.ncbi.nlm.nih.gov/pmc/articles/PMC5830125/> [↑](#footnote-ref-3)
4. E-Bug. (2019). E-Bug Peer Educator Training Manual. Page 12. Available at: <https://www.e-bug.eu/peereducation/download/english/Science%20Show%20Peer%20Education.pdf> [↑](#footnote-ref-4)
