# Supplementary material for: Development of health information materials on antimicrobial resistance with lay workers in Grahamstown/Makhanda, South Africa
Source: Front Public Health. 2025 Oct 29;13:1542448. doi: 10.3389/fpubh.2025.1542448 (PMC12608051; doi:10.3389/fpubh.2025.1542448)
Supplement: Supplementary file 2 [file Data_Sheet_2.docx]

**Appendix 1 – Drafts of the AMR HIL**

*Table S1: Demographics of the study participants*

| *Participant Number* | *Age* | *Gender* | *Home Language* | *Profession* |
| --- | --- | --- | --- | --- |
| *1* | *41* | *Female* | *Afrikaans* | *CHW* |
| *2* | *55* | *Female* | *IsiXhosa* | *PA* |
| *3* | *30* | *Female* | *IsiXhosa* | *CHW* |
| *4* | *44* | *Female* | *IsiXhosa* | *PA* |
| *5* | *33* | *Female* | *IsiXhosa* | *PA PB* |
| *6* | *36* | *Female* | *IsiXhosa* | *CHW* |
| *7* | *33* | *Male* | *IsiXhosa* | *CHW* |
| *8* | *50* | *Female* | *IsiXhosa* | *CHW* |
| *9* | *59* | *Female* | *IsiXhosa* | *PA* |
| *10* | *31* | *Female* | *IsiXhosa* | *PA* |
| *11* | *30* | *Female* | *IsiXhosa* | *CHW* |
| *12* | *39* | *Female* | *IsiXhosa* | *PA* |
| *13* | *29* | *Female* | *IsiXhosa* | *CHW* |
| *14* | *55* | *Female* | *IsiXhosa/ English* | *PA* |
| *15* | *46* | *Female* | *IsiXhosa* | *CHW* |

Table S2. Demographics for Rhodes University peer educators attending the health information leaflet about antimicrobial resistance (AMR HIL) workshop on 23 May 2019.

| *Participant Number* | *Age* | *Gender* | *Home Language* |
| --- | --- | --- | --- |
| *1* | *59* | *Male* | *IsiXhosa* |
| *2* | *51* | *Female* | *IsiXhosa* |
| *3* | *50* | *Female* | *IsiXhosa* |
| *4* | *50* | *Male* | *IsiXhosa* |
| *5* | *56* | *Female* | *IsiXhosa* |
| *6* | *45* | *Female* | *IsiXhosa* |
| *7* | *44* | *Female* | *IsiXhosa* |
| *8* | *36* | *Female* | *IsiXhosa* |
| *9* | *33* | *Female* | *IsiXhosa* |
| *10* | *42* | *Female* | *IsiXhosa* |
| *11* | *26* | *Female* | *IsiXhosa* |
| *12* | *48* | *Male* | *IsiXhosa* |
| *13* | *55* | *Female* | *IsiXhosa* |
| *14* | *40* | *Female* | *IsiXhosa* |
| *15* | *42* | *Female* | *IsiXhosa* |
| *16* | *51* | *Female* | *IsiXhosa* |
| *17* | *49* | *Female* | *IsiXhosa* |
| *18* | *53* | *Male* | *IsiXhosa* |
| *19* | *57* | *Female* | *IsiXhosa* |
| *20* | *49* | *Female* | *English* |
| *21* | *57* | *Female* | *IsiXhosa* |

*Table S3. Demographics for Rhodes University peer educators attending the AMR HIL on 23 October 2019.*

| *Participant Number* | *Age* | *Gender* | *Home Language* | *Education Level* | *Job Title* | *Institution/ Clinic* |
| --- | --- | --- | --- | --- | --- | --- |
| *1* | *51* | *Female* | *IsiXhosa* | *Grade 11* | *Room attendant* | *Rhodes University* |
| *2* | *58* | *Female* | *IsiXhosa* | *Grade 10* | *Cleaner* | *Rhodes University* |
| *3* | *40* | *Female* | *IsiXhosa* | *Grade 12* | *Housekeeper* | *Rhodes University* |
| *4* | *45* | *Female* | *IsiXhosa* | *Grade 12* | *Housekeeper* | *Rhodes University* |
| *5* | *46* | *Female* | *IsiXhosa* | *Grade 12* | *Office administrator* | *Rhodes University Health Care Centre* |
| *6* | *36* | *Female* | *IsiXhosa* | *Diploma* | *Housekeeper* | *Rhodes University* |
| *7* | *45* | *Female* | *IsiXhosa* | *Grade 12* | *Cleaner* | *Rhodes University* |

**Appendix 2 – Drafts of the AMR HIL**

*Table S4: Table of changes from draft 1 to draft 2 of the health information leaflet about antimicrobial resistance (AMR HIL).*

|  | *Change Required* | *Change Made* |
| --- | --- | --- |
| *S1* | *Change the following picture to avoid the depiction that all antimicrobials green and white capsules:*  *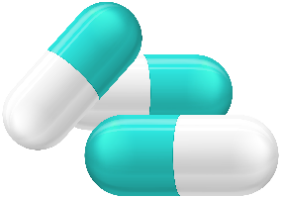* | *The picture was changed to:*  *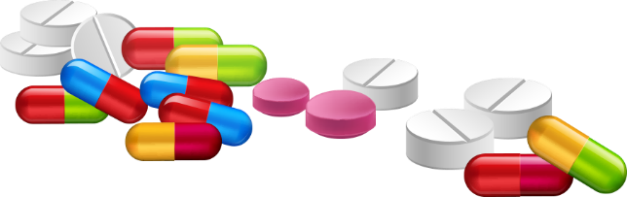* |
| *S1, S2* | *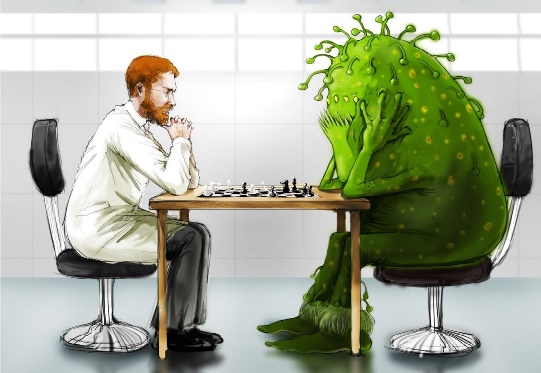Change the following picture, as it may be depicted wrongly:* | *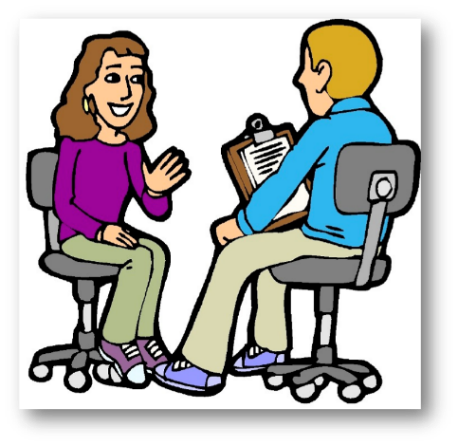Picture changed to:*  *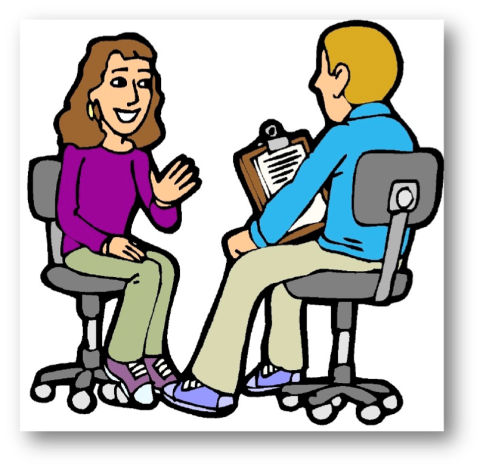* |
| *S1* | *Use the term ‘appropriate antimicrobials’ and rephrase the following sentence:*  *Antimicrobials are often used to treat these infections.* | *The sentence was rephrased to:*  *These infections are treated with appropriate antimicrobials.* |
| *S1* | *Change the following picture, as its placement is wrong:* 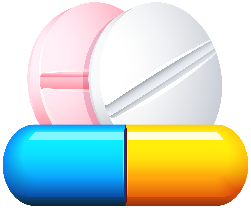 **Avoid overuse** | *The picture was changed to:* 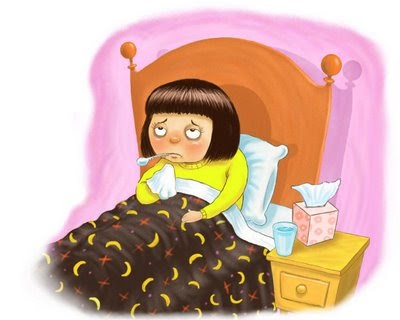 **Infected patient** |
| *S1* | *Replace the word ‘happens’ with ‘is’ and use the term ‘appropriate antimicrobials’ in the following sentence:*  *Antimicrobial Resistance happens when microorganisms no longer respond to the antimicrobials used to treat infections.* | *The sentence was rephrased to:*  *Antimicrobial Resistance is when microorganisms no longer respond to the appropriate antimicrobials used to treat infections.* |
| *S1* | *Replace the word ‘sex’ with ‘gender’ in the following sentence:*  *Antimicrobial Resistance can affect any race, sex or age.* | *The sentence was rephrased to:*  *Antimicrobial Resistance can affect any race, gender or age.* |
| *S1* | *Change the following picture to be more context specific, and to avoid the depiction that all antimicrobials are red and white capsules:* 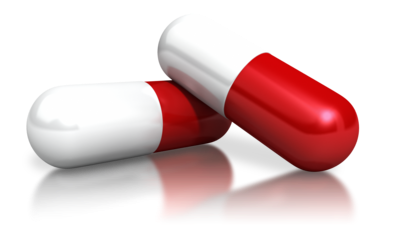 **Antimicrobials** | *The picture was changed to:* 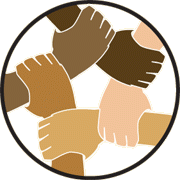 **All races are affected** |
| *S3* | *State whether the following statistic is global or local:*  *Antimicrobial Resistance currently causes 700 000 deaths per year.* | *The sentence was rephrased to:*  *Antimicrobial Resistance currently causes 700,000 deaths per year worldwide.* |
| *S3* | *State whether the following statistic is global or local:*  *By 2050, Antimicrobial Resistance will cause up to 10 million deaths per year.* | *The sentence was rephrased to:*  *By 2050, Antimicrobial Resistance will cause up to 10 million deaths per year worldwide, out of which 4,150,000 deaths will be in Africa.* |
| *S1* | *State the number to deaths which will occur locally to make the following sentence more context specific:*  *By 2050, Antimicrobial Resistance will cause up to 10 million deaths per year.* | *Please note that only African statistics were available for this data. The sentence was rephrased to:*  *By 2050, Antimicrobial Resistance will cause up to 10 million deaths per year worldwide, out of which 4,150,000 deaths will be in Africa.* |
| *S4* | *Replace the words ‘low- and middle-income countries’ to ‘developing countries’ in the following sentence, for lower literacy purposes:*  *Low- and middle-income countries are most vulnerable to Antimicrobial Resistance.* | *The sentence was rephrased to:*  *Developing countries are more at risk to Antimicrobial Resistance.* |
| *S3* | *Replace the word ‘vulnerable’ in the following sentence, for lower literacy purposes:*  *Low- and middle-income countries are most vulnerable to Antimicrobial Resistance.* | *The sentence was rephrased to:*  *Developing countries are more at risk to Antimicrobial Resistance.* |
| *S1* | *Change the word ‘Overprescribing’ to ‘Inappropriate use’ in the following sentence:*  *Overprescribing e.g. antibiotics for viral infections (colds and flu)* | *The sentence was rephrased to:*  *Inappropriate use e.g. use of antibiotics for viral infections (colds and flu)* |
| *S1* | *Mention ‘livestock’ before ‘fish farming’, as it is more common in South Africa, and give examples of ‘livestock’ in the following sentence:*  *Overuse in fish farming and livestock* | *The sentence was rephrased to:*  *Overuse in livestock (e.g. cattle, goat, pig, chicken) and fish farming* |
| *S1, S4* | *Change the following picture to be more context specific:* 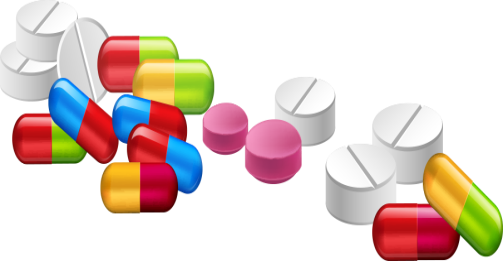 **Overuse** | *The picture was changed to:*  *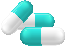* 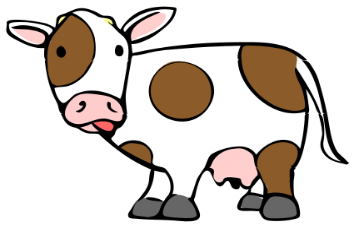 **Overuse in livestock** |
| *S1* | *‘Sharing’ and ‘Using leftovers’ of what? Please clarify.* | *The bullet points were combined and rephrased to:*  *Sharing and using leftover antimicrobials* |
| *S1* | *‘Poor hygiene’ is not clear and does not fit under the ‘Environment’ subheading* | *The subheading was rephrased to ‘Environment and hygiene’, and ‘poor hygiene’ was removed as a bullet point* |
| *1* | *State who is being referred to in the following bullet point:*  *Not washing hands regularly* | *The bullet point was rephrased to:*  *Irregular washing of hands by the population* |
| *S1, S4* | *Replace/delete the following picture, as it may be depicted wrongly:* 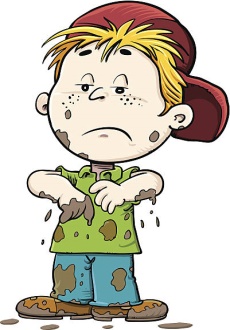 **Poor hygiene** | *The picture was deleted.* |
| *S4* | *Replace the word ‘Consequences’ in the following heading for lower literacy purposes:*  *Consequences of Antimicrobial Resistance* | *The heading was rephrased to:*  *Impact of Antimicrobial Resistance on the Healthcare System and Overall Population* |
| *S1* | *Clarify who the diagram has an impact on* | *The heading above the diagram was rephrased to:*  *Impact of Antimicrobial Resistance on the Healthcare System and Overall Population* |
| *S1* | *The wording ‘death rates and tolls’ is not clear in the following sentence in the diagram:*  *Higher death rates and tolls* | *The sentence in the diagram was rephrased to:*  *Higher death rates* |
| *S4* | *Rearrange the arrows in the following diagram, as one does not always lead to the next:*  *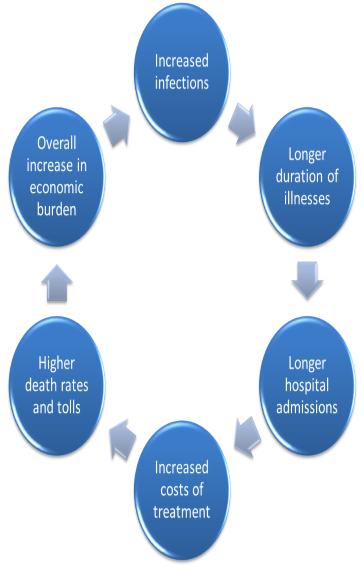* | *The arrows in the diagram were rearranged as follows:*  *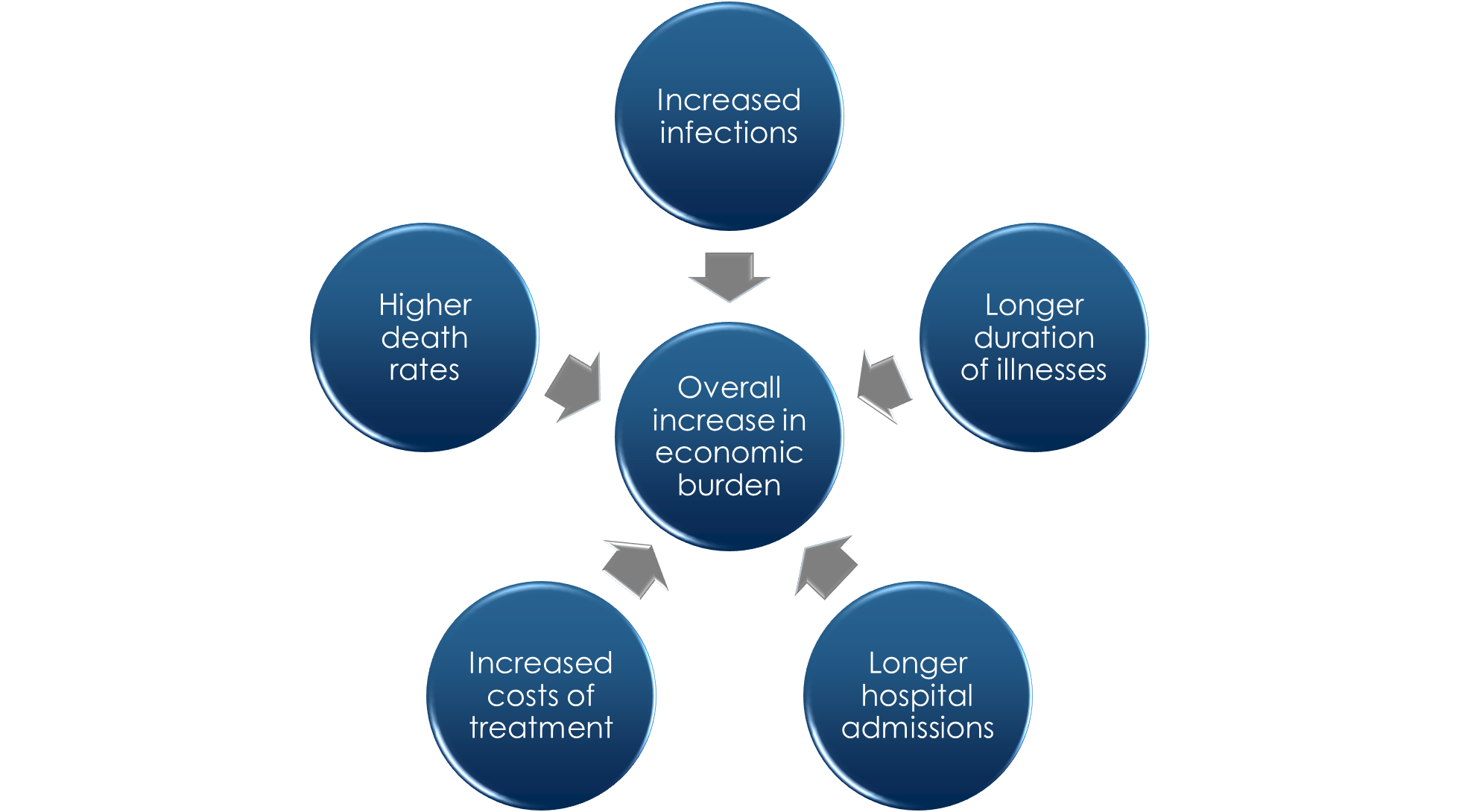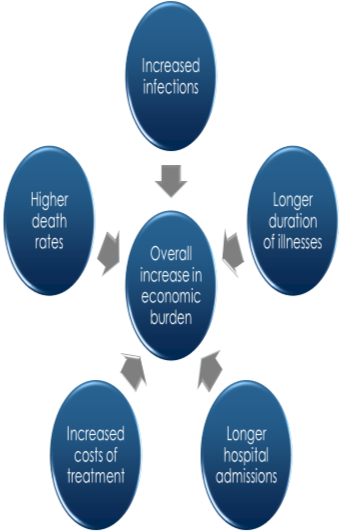* |
| *S1* | *Rephrase the following heading to avoid the use of a second person:*  *How Can You Prevent Antimicrobial Resistance?* | *The heading was rephrased to:*  *Prevention of Antimicrobial Resistance* |
| *S5* | *Change the caption of the following picture, as both, doctors and nurses prescribe in the public sector:*   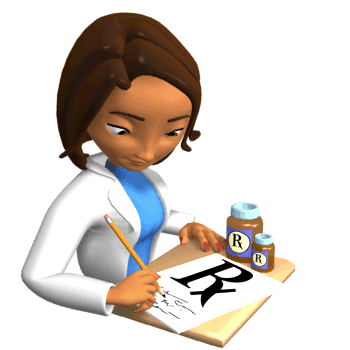 **Doctor prescribing** | *The caption of the picture was changed to:* 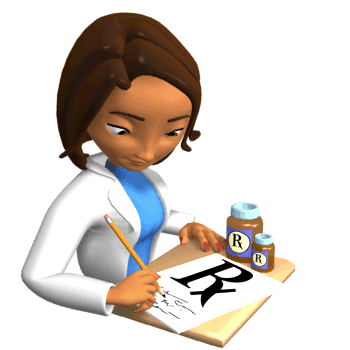 **Prescribers** |
| *S4* | *Add information about missing a dose of antimicrobials under the ‘Preventions’ heading* | *The information was added, and the following sentence was rephrased to:*  *Always complete the treatment course and do not miss doses, even if one feels better* |
| *S1* | *Rephrase the following sentence to avoid the use of a second person:*  *Always complete the treatment course, even if you feel better* | *The sentence was rephrased to:*  *Always complete the treatment course and do not miss doses, even if one feels better* |
| *S1* | *Who should ‘never share antimicrobials’? Please clarify.* | *The sentence was rephrased to:*  *Never share antimicrobials with others* |
| *S1* | *‘Never use leftovers’ of what? Please clarify.* | *The sentence was rephrased to:*  *Never use leftover antimicrobials from previously treated infections* |
| *S1* | *Replace/delete the picture below, as it may depict wrongly:* 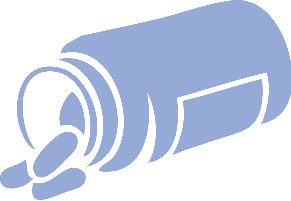 **Never use leftovers**    *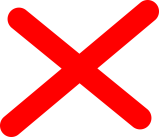* | *The picture was deleted.* |
| *S6* | *Delete the ‘Prevent infections by:’ subheading for continuous flow of related information* | *The subheading was deleted* |
| *S4* | *Add ‘with clean water and soap’ to the following phrase:*  *Regularly washing your hands* | *Information added and phrased rephrased to:*  *Wash hands regularly with clean water and soap* |
| *S1, S5* | *Rephrase the following phrase, as it is not applicable to health care professionals and providers:*  *Avoiding contact with sick people* | *Phrase rephrased to:*  *Take precautions when coming into contact with sick people* |
| *S5* | *Include the word ‘family’ in the following phrase, to include children as well as adults:*  *Keeping up-to-date with your vaccinations* | *The phrase was rephrased to:*  *Keep the family up-to-date with vaccinations* |

*Table S5: Table of changes from draft 2 to draft 3 of the health information leaflet about antimicrobial resistance (AMR HIL).*

|  | *Change Required* | *Change Made* |
| --- | --- | --- |
| *1* | *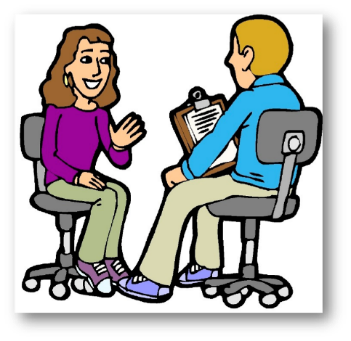Change the following picture, as it may be depicted wrongly:* | *The picture was changed to:*  *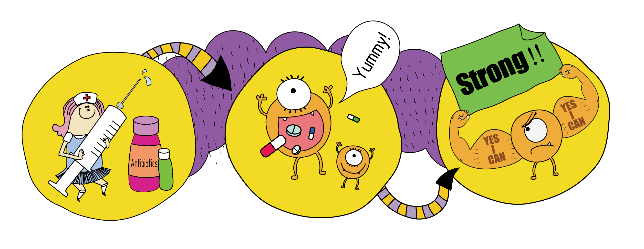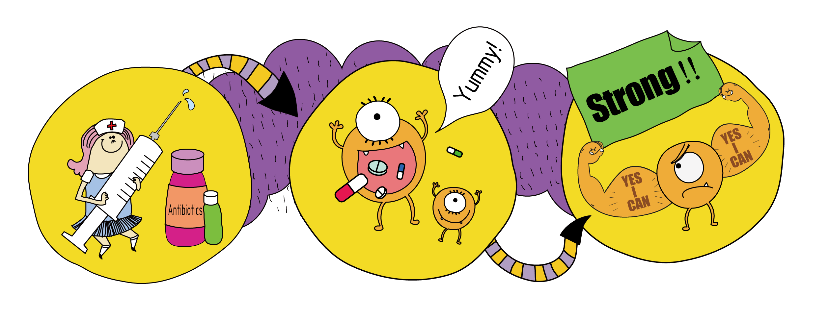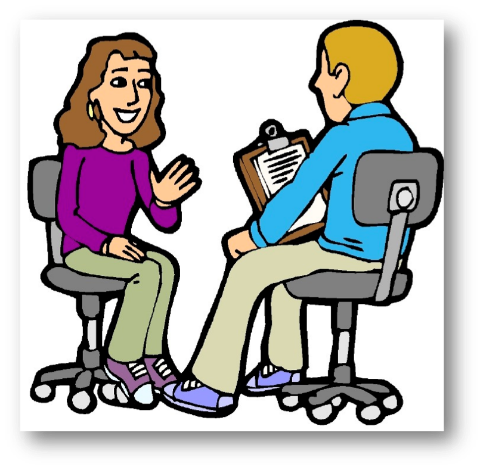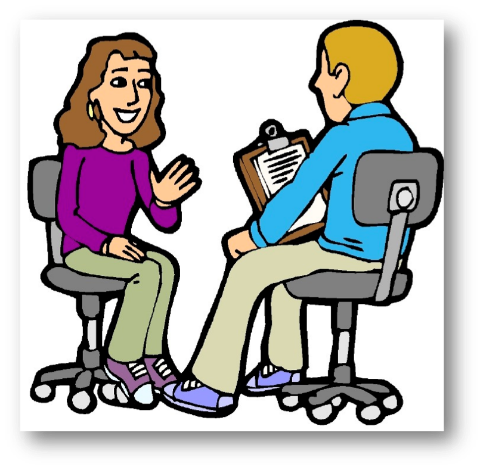* |
| *2* | *Rephrase/delete the following sentence:*  *“Microorganisms such as bacteria, viruses, fungi and parasites cause infections. These infections are treated with appropriate antimicrobials.”* | *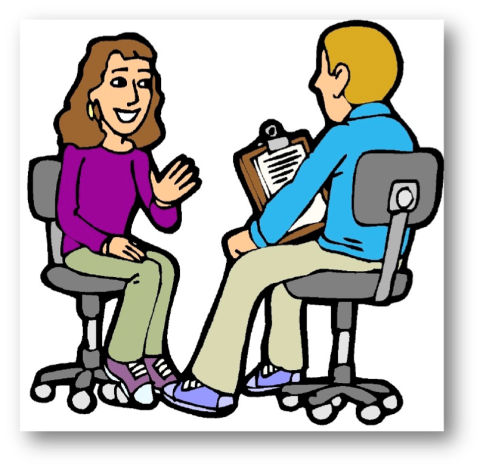The sentence was deleted.* |
| *3* | *Rephrase the following sentence:*  *“Antimicrobial Resistance is when microorganisms no longer respond to the appropriate antimicrobials used to treat infections.”* | *The sentence was rephrased to:*  *“Antimicrobial Resistance is when microbes (e.g. bacteria and viruses) no longer respond to the appropriate medicines used to treat infections.”* |
| *4* | *Replace/delete the following picture, as its placement is wrong:* 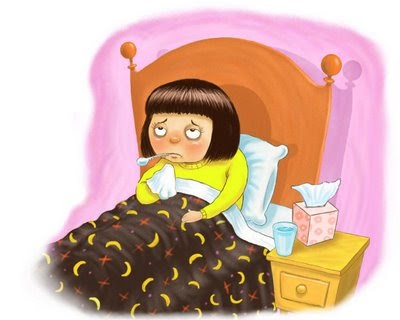 **Infected patient** | *The picture was deleted.* |
| *5* | *Replace the word ‘gender’ with ‘sex’ in the following sentence:*  *“Antimicrobial Resistance can affect any race, gender or age.”* | *The sentence was rephrased to:*  *“Antimicrobial Resistance can affect any race, sex or age.”* |
| *6* | *Change the placement of the following section:*  *“DID YOU KNOW?*   - *Antimicrobial Resistance currently causes 700,000 global deaths per year.* - *By 2050, Antimicrobial Resistance will cause up to 10 million global deaths per year, out of which 4,150,000 deaths will be in Africa.* - *Developing countries are more at risk to Antimicrobial Resistance.”* | *This section was moved to the back panel of the HIL.* |
| *7* | *Change the following heading to a question format:*  *“Causes of Antimicrobial Resistance”* | *The heading was changed to:*  *“What causes Antimicrobial Resistance?”* |
| *8* | *Rephrase the following subheading for more clarity:*  *“Overuse of antimicrobials:”* | *The subheading was rephrased to:*  *“Overuse of antibiotics by prescribers and patients:”* |
| *9* | *Rephrase and further elaborate on the following bullet point:*  *“Inappropriate use e.g. use of antibiotics for viral infections (colds and flu)”* | *The bullet point was rephrased to:*   - *“Using antibiotics when they are not necessary* - *Antibiotics fight infections caused by bacteria. Antibiotics do not work against viruses such as colds and flu”* |
| *10* | *Delete the following bullet point, as it is not context-appropriate:*  *“Overuse in livestock (e.g. cattle, goat, pig, chicken) and fish farming”* | *The bullet point was deleted.* |
| *11* | *Replace/delete the following picture as it is not context-appropriate:*  *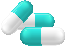* 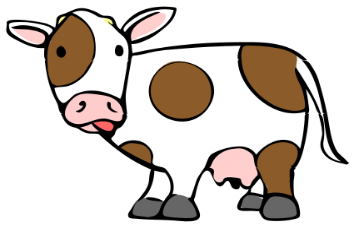 **Overuse in livestock** | *The picture was changed to:* 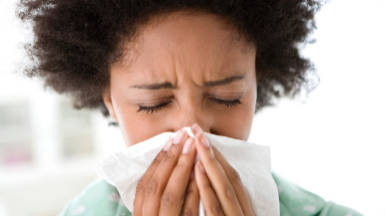 **Colds and flu (virus)** |
| *12* | *Rephrase the following subheading for more clarity:*  *“Misuse of antimicrobials:”* | *The subheading was rephrased to:*  *“Misuse of antibiotics by patients:”* |
| *13* | *Rephrase the following bullet point for personalization:*  *“Not finishing the treatment course”* | *The bullet point was rephrased to:*  *“Not finishing your course of antibiotics”* |
| *14* | *Separate the following bullet points into two bullet points:*  *“Sharing and using leftover antimicrobials”* | *The bullet point was separated into two bullet points:*   - *“Sharing antibiotics with others* - *Using leftover antibiotics”* |
| *15* | *Change the word ‘antimicrobials’ to ‘antibiotics’ in the following picture’s caption:* 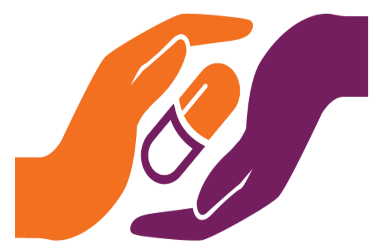 **Sharing antimicrobials** | *Caption changed to:* 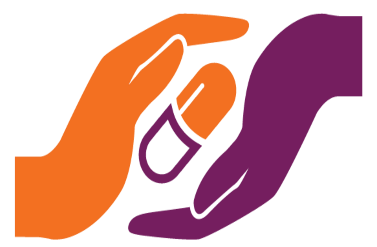 **Sharing antibiotics** |
| *16* | *Rephrase the following subheading to make it more context-appropriate:*  *“Environment and hygiene:”* | *The subheading was changed to:*  *“Lack of hygiene:”* |
| *17* | *Replace/delete the following bullet point as it is not context-appropriate:*  *“Poor infection control in hospitals and clinics”* | *The bullet point was deleted, and the following bullet point was added:*  *“Keeping a place dirty”* |
| *18* | *Rephrase the following bullet point:*  *“Irregular washing of hands by the population”* | *The bullet point was rephrased to:*  *“Not washing your hands regularly”* |
| *19* | *Add an appropriate picture to support the information regarding hygiene.* | *A picture was added:* 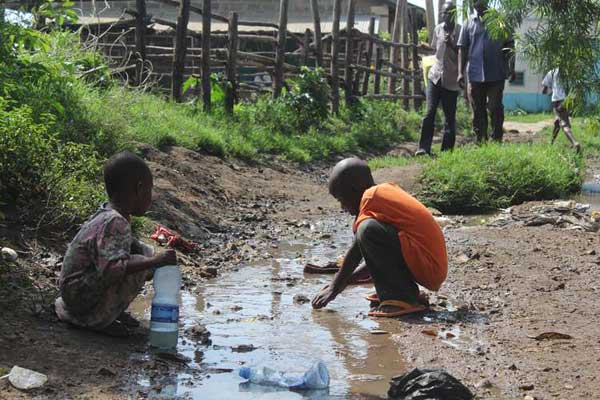 **Lack of hygiene** |
| *20* | *Replace/delete the following subheading and bullet point as it is not context-appropriate:*  *“Research and development:*   - *Lack of development of new antimicrobials”* | *The subheading and bullet point were deleted.* |
| *21* | *Change the format of the following diagram as it may be misinterpreted with the arrows and flow of information:*  *Impact of Antimicrobial Resistance on the Healthcare System and Overall Population*  *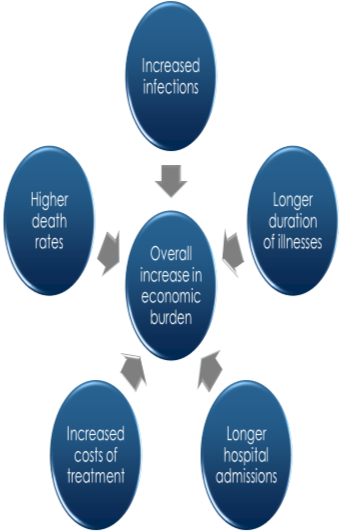* | *The information was changed from a diagram format to a bullet point format and moved to the first panel of the HIL:*  *“What is the Impact of Antimicrobial Resistance?*     - *Increased infections* - *Longer duration of illnesses* - *Longer hospital admissions* - *Increased costs of treatment* - *Higher death rates”* |
| *22* | *Add information on hand hygiene to the HIL – why and when should you wash your hands?* | *A section on hand hygiene was added:*  *“Why Should You Wash Your Hands?*   - *To remove harmful microbes from your hands and places* - *To reduce getting infections* - *To prevent the spread of infections* - *Washing your hands with soap and water is better than washing your hands with water alone*   *When Should You Wash Your Hands?*   - *Before, during and after preparing food, especially raw meat* - *After using the toilet* - *After touching animals and animal waste* - *After coughing, sneezing or blowing your nose* |
| *23* | *Add an appropriate picture to support the information regarding hand hygiene.* | *A picture was added:* 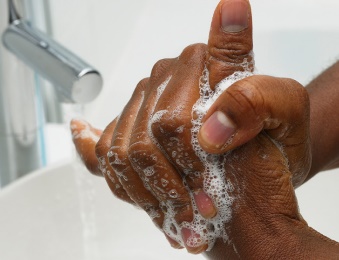 **Wash hands with soap** |
| *24* | *Add an appropriate picture to support the information regarding hand hygiene.* | *A picture was added:* 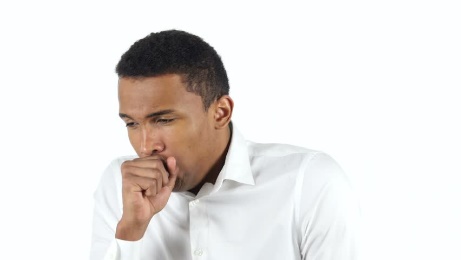 **Coughing** |
| *25* | *Change the following heading to a question format:*  *“Prevention of Antimicrobial Resistance”* | *The heading was changed to:*  *“How Can You Prevent Antimicrobial Resistance?”* |
| *26* | *Add a subheading to clarify and breakdown the information.* | *The following subheading was added:*  *“To reduce changes of getting infected, you should:”* |
| *27* | *Rephrase the following bullet point:*  *“Keep the family up-to-date with vaccinations”* | *The bullet point was rephrased to:*  *“Keep up-to-date with vaccinations”* |
| *28* | *Rephrase the following bullet point:*  *“Only use antimicrobials when prescribed by a certified health care professional”* | *The bullet point was rephrased to:*  *“Only use antibiotics when prescribed by a doctor or nurse”* |
| *29* | *Rephrase the following bullet point:*  *“Always complete the treatment course and do not miss doses, even if one feels better”* | *The bullet point was separated into two bullet points, and rephrased to:*   - *“Always finish your treatment course, even if you feel better* - *Do not miss any doses”* |
| *30* | *Rephrase the following bullet point:*  *“Never share antimicrobials with others”* | *The bullet point was rephrased to:*  *“Do not share antibiotics with others”* |
| *31* | *Rephrase the following bullet point:*  *“Never use leftover antimicrobials from previously treated infections”* | *The bullet point was rephrased to:*  *“Never use leftover antibiotics”* |
| *32* | *Rephrase/delete the following bullet point”*  *“Take precautions when coming into contact with sick people”* | *The bullet point was deleted.* |
| *33* | *Add a bullet point regarding double doses.* | *The following bullet point was added:*  *“Do not take double doses when you miss a dose”* |
| *34* | *Change/delete the following picture, as it may be depicted wrongly:* 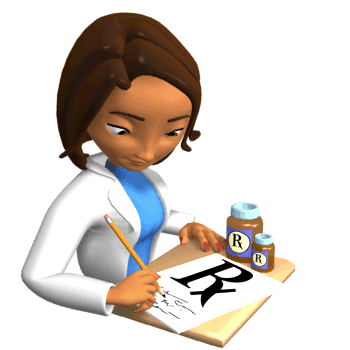 **Prescribers** | *The picture was deleted.* |
| *35* | *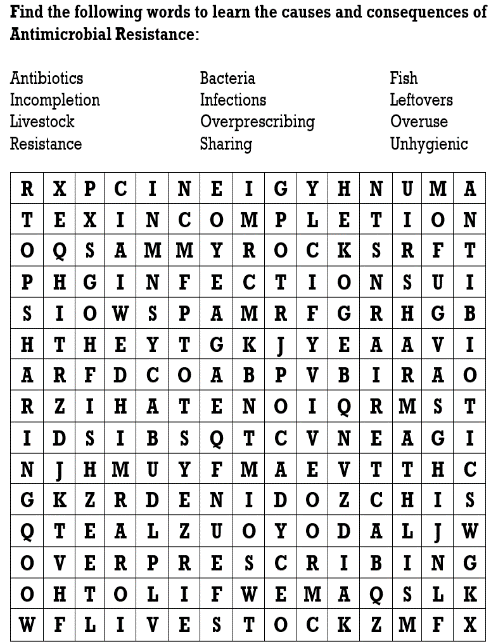Delete the following crossword as it is not appropriate for the target population:* | *The crossword was deleted.* |
| *36* | *Add a picture on the back panel in place of the crossword.* | *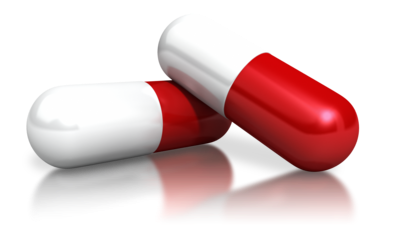A picture was added on the back panel:* |
